# Agent-Based Simulation for Seasonal Guinea Worm Disease in Chad Dogs

**DOI:** 10.4269/ajtmh.19-0466

**Published:** 2020-09-08

**Authors:** Tyler Perini, Pinar Keskinocak, Zihao Li, Ernesto Ruiz-Tiben, Julie Swann, Adam Weiss

**Affiliations:** 1 Georgia Institute of Technology, Atlanta, Georgia;; 2 The Carter Center, Atlanta, Georgia;; 3 North Carolina State University, Raleigh, North Carolina

## Abstract

The campaign to eradicate dracunculiasis (Guinea worm [GW] disease) and its causative pathogen *Dracunculus medinensis* (GW) in Chad is challenged by infections in domestic dogs, which far outnumber the dwindling number of human infections. We present an agent-based simulation that models transmission of GW between a shared water source and a large population of dogs. The simulation incorporates various potential factors driving the infections including external factors and two currently used interventions, namely, tethering and larvicide water treatments. By defining and estimating infectivity parameters and seasonality factors, we test the simulation model on scenarios where seasonal patterns of dog infections could be driven by the parasite’s life cycle alone or with environmental factors (e.g., temperature and rainfall) that could also affect human or dog behaviors (e.g., fishing versus farming seasons). We show that the best-fitting model includes external factors in addition to the pathogen’s life cycle. From the simulation, we estimate that the basic reproductive number, *R*_0_, is approximately 2.0; our results also show that an infected dog can transmit the infection to 3.6 other dogs, on average, during the month of peak infectivity (April). The simulation results shed light on the transmission dynamics of GWs to dogs and lay the groundwork for reducing the number of infections and eventually interrupting transmission of GW.

## INTRODUCTION

The campaign to eradicate Guinea worm (GW) disease in humans has made much progress since 1986 when the annual burden of the disease in 21 countries (19 countries of the African Sahel, plus India and Pakistan in Asia) was estimated to be 3.5 million cases. During 2018, only 28 human cases were reported worldwide: 17 from Chad, 10 from South Sudan, and one from Angola. However, Chad also reported 1,040 domestic dogs with GW infections during 2018.^1^

*Dracunculus medinensis*, more commonly referred to as GW, is a parasitic nematode. The GW’s definitive host (e.g., humans and dogs) becomes infected via ingestion of fresh water copepods (the intermediate host) harboring infective GW third-stage larvae, referred to as L3s, for example, consumed through drinking water or improperly cooked/cured aquatic animals harboring L3s in their somatic tissue.† After mating in the definitive host, a gravid female worm emerges from the host’s skin after about a year. While emerging, if the worm is submerged in a source of water, the worm releases tens of thousands of first-stage larvae, some of which are ingested by copepods, thus restarting the life cycle.

Human cases of GW disease were confirmed in Chad during 2010, 10 years after the last reported human infection. At the request of Chad’s government, the Carter Center supported the Chad Guinea Worm Eradication Program (GWEP‡) to be relaunched in April 2012, including an active village-based surveillance system in about 700 villages and the investigation of reports of alleged cases for information leading to confirmation of cases of GW disease. Whereas human infections remained low (less than 20 per year), dog infections doubled annually, reaching 1,011 cases across 271 villages in 2016. By the end of 2017, the Chad GWEP was monitoring human and animal GW infections in 1,860 villages.^6^ To date, infections in dogs are not fully understood.

We develop a detailed simulation model to capture the dynamics of GW transmission in dogs, considering the GW life cycle, seasonality, and interventions. Computer simulation models have been used to understand the epidemiology of diseases such as influenza,^7,8^ HIV/AIDS,^9^ and malaria,^10,11^ among others. Few publications include mathematical models on GW infections; these are mostly deterministic compartmental models, do not always use empirical data for calibration and validation^12–14^ or consider only human hosts, and do not capture the dynamics of transmission in dogs, which is paramount in the current epidemic in Chad.^15–17^

The model by Ghosh et al.^14^ includes a representation of a dog population and accounts for annual infection rates for both dogs and humans. Their results suggest that dogs are more important for the continued propagation of GWs in Chad than humans. However, their model does not capture the significant seasonal dynamics of GW transmission. In this article, we present a stochastic agent-based simulation model for GW transmission in dogs, incorporating seasonality and interventions into the model.

Historical data indicate seasonality for GW infections,^18,19^ including in the recent cases of Chad dogs: it has been observed that peak GW transmission in humans occurs during the rainy season (May to August) in the Sahelian zone, whereas peak transmission occurs during the dry season (September to January) in the humid savanna.^20,21^ Although infection data and expert field experience support these observations for human infections, the literature connecting environment and transmission is outdated and neither focuses on Chad nor dog behaviors.^21^§ Even mathematical models that include a seasonality component do so in an “arbitrary” fashion, that is, without specific environmental data. Environment-driven seasonality has been found to be critical to many diseases, including influenza,^22^ malaria,^23^ and cholera.^24^ We hypothesize that GW transmission is driven in large part by environmental patterns, which directly or indirectly affects the behaviors of humans and dogs as well as the life cycle or availability of the parasite or other hosts (e.g., intermediate or paratenic). We calibrate our model using data from the Chad GWEP and monthly environmental data in Chad, and we provide with plausible explanations for observed patterns in dog infections.

Overall, our model provides a general framework for studying GW transmission in dogs; it can be used to test various hypothetical scenarios, contributes to the understanding of the disease, and lays the groundwork for future studies, including, most pressingly, a formal assessment and prioritization of intervention strategies.

## MATERIALS AND METHODS

### Data.

**Figure 1.**
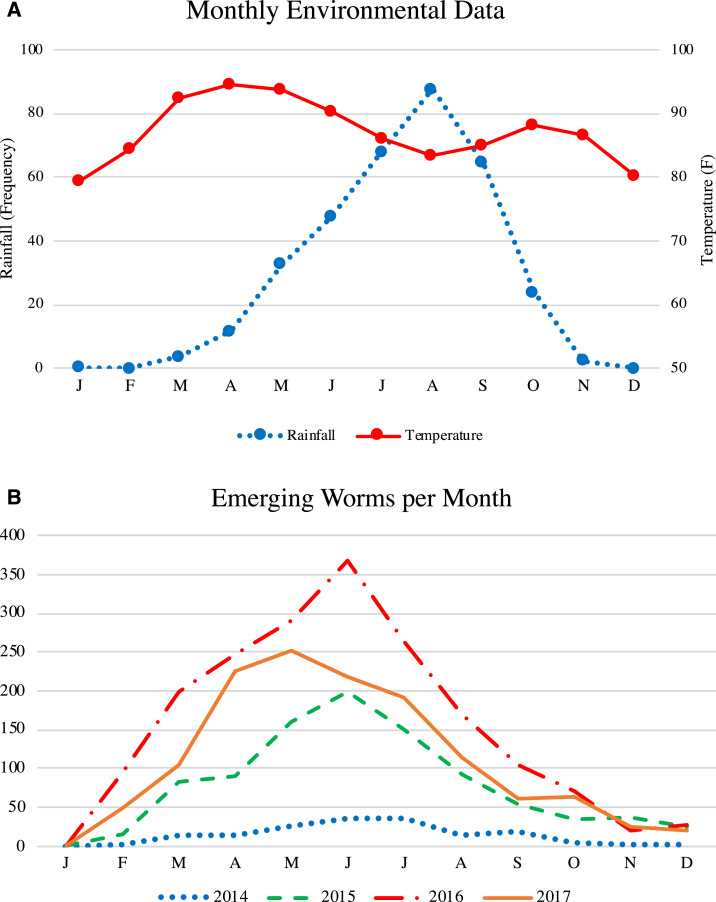
Data used in the simulation model. (**A**) averages of 2013–2017 environmental data. (**B**) worm emergence from 2014 to 2017 used for initialization and parameter calibration. This figure appears in color at www.ajtmh.org.

Environmental data on daily precipitation and temperature measurements were collected from 17 weather stations in Chad.^25^ We used the average monthly rainfall and temperature over the years 2013–2017; both are illustrated in Figure 1A (see Supplemental Appendix Section 1.1 for more details). Note that the rainfall peaks in August, and the temperature distribution is bimodal with a “heat wave” before and after the rainy season.

Infection data were provided by the Chad GWEP, including the number of worms emerging from dogs per month and the number of dogs with an emerging worm per month for the years 2014–2017; the former is illustrated in Figure 1B. It is observed that dog infections consistently peak in May or June, that is, before the peak of the rainy season in Chad. In addition, the Chad GWEP reported data for intervention coverage: the proportion of infected dogs contained via tethering and the proportion of Abate applications made in response to water contamination events per year during years 2014–2017.^26^

### Simulation model.

To estimate the prevalence of dog infections over time and to understand transmission dynamics, we developed a stochastic agent-based simulation. Unlike a compartmental model, such as a susceptible-infected-susceptible model, the stochastic agent-based simulation model tracks the activities, behaviors, and health status of participants (e.g., dogs) in the system at the individual level, capturing variations and incorporating complex interactions with transmission sources, interventions, and/or treatments.^27,28^ For example, more than one worm may be consumed by or emerge from a dog, separated in time, representing multiple, overlapping infections. In addition, an agent-based model provides a framework for future analyses with even greater complexity.

**Figure 2.**
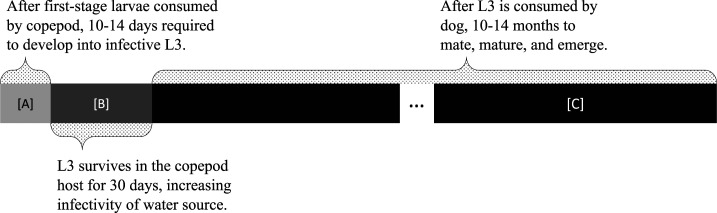
Relevant time durations for Guinea worm life cycle in the model.

**Figure 3.**
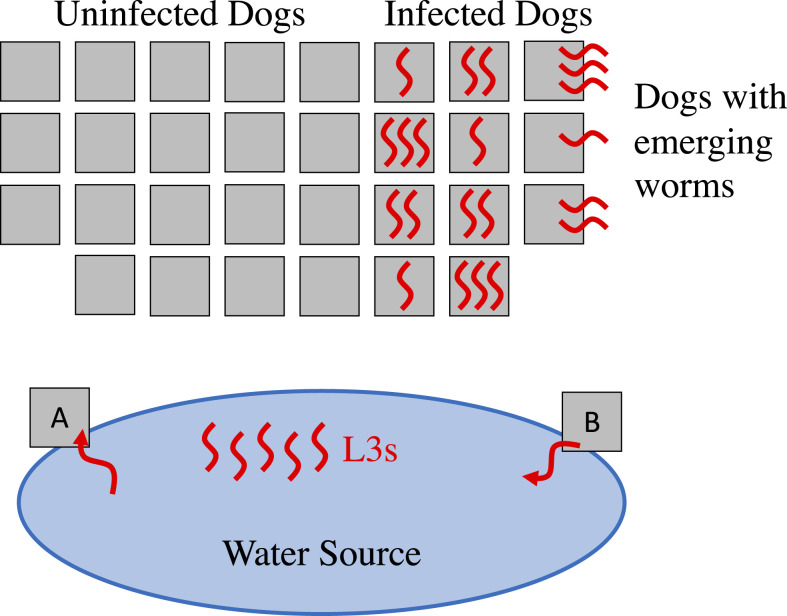
Simulation represents dogs, worms, and a single water source. Because dogs interact with the water source, they acquire infection with some probability (**A**), and dogs with emerging worms increase the infectivity of the water source (**B**). This figure appears in color at www.ajtmh.org.

The model simulates the life cycle of GWs, including pertinent time periods (summarized in Figure 2), along with daily interactions between the dogs, worms, and water source over multiple years. The model is illustrated in Figure 3; see Supplemental Appendix Section 1 for details.

### Intervention methods.

The simulation incorporates two existing/current interventions, 1) and 2) given as follows, and a third generalized intervention, 3) (see Supplemental Appendix Section 1.6 for details).

1. Tethering: Dogs are kept on a leash for 30 days after the GW begins emergence to prevent the dog from spreading GW larva and are provided safe food and water. In the simulation, dogs are tethered with some probability, and we assume that tethered dogs do not acquire new infections during this time.**
2. Abate treatment: An organophosphate larvicide temephos (ABATE Larvicide, BASF [Ludwigshafen, Germany], or just “Abate”) paralyzes, thus eventually killing, copepods in the water; once applied, it is effective for up to 30 days. In the simulation, this intervention has two effects: it eliminates a proportion of the number of infected copepods (and the corresponding L3s) in the water source, thereby reducing infectivity of the water source, and it protects a proportion of the dogs from consuming L3s, where the proportion of protected dogs depends on the reported usage of Abate.
3. Other interventions: This generic category represents the combined benefits of various efforts to prevent dogs from consuming L3s, including public education efforts, providing safe water to dogs, and preventing dogs from eating fish entrails during food preparation (usually by burying); because the coverage level is unknown, its parameter values (one per year) are calibrated along with other parameters in the simulation.

### Factors driving seasonality.

**Figure 4.**
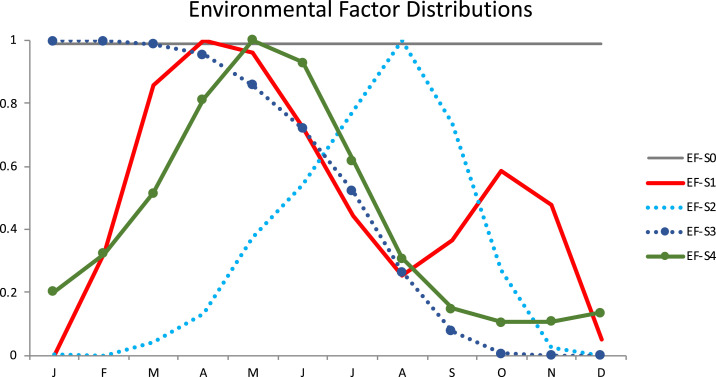
Environmental factor scenarios plotted for comparison. EF-S0 is constant. EF-S1 is the normalized average monthly temperature. EF-S2 is the normalized average monthly rainfall, and EF-S3 is one minus the cumulative rainfall. EF-S4 peaks between the first heat wave and peak rainfall. This figure appears in color at www.ajtmh.org.

Dog infections exhibit seasonality (Figure 1B), which could be directly because of environmental factors (EFs) or indirectly because of modified human or dog behaviors. In the simulation, we include 12 parameters (1 per month), which we call the EF coefficients. A lower EF coefficient in a given month results in a lower infectivity of the water source in the simulation. We test the following five hypothetical EF scenarios, as depicted in Figure 4 (see Supplemental Appendix Section 1.8 for details, including exact formulas). Note that we test several different hypothetical scenarios to cover a wide variety of potential explanations.

1. EF-S0: External seasonality has no effect on infectivity, that is, seasonality is driven solely by the life cycle of GWs.
2. EF-S1: Infectivity increases as temperature increases, for example, because of the higher likelihood of dogs to drink from potentially infected water sources or the increase in the availability of hosts (e.g., copepods).
3. EF-S2: Infectivity increases as rainfall increases, which may be related to the availability of hosts.
4. EF-S3: Infectivity decreases as annual rainfall accumulates, for example, because of the decrease in the density of L3s in water sources or change in the availability of other hosts, which is supported by data in Hunter.^29^
5. EF-S4: Infectivity is greatest before the rainy season and when temperature is high, which combines EF-S1 and EF-S3. The water sources are likely to be most infective in the months before the peak of the rainy season (before water bodies have gotten too large), and dogs are more likely to drink infected water after the largest heat wave, which is consistent with discussions in Watts^21^†† and Hunter.^29^‡‡

### Parameter calibration.

We compute the error for the number of worms emerging per month and the number of dogs with emerging worms per month as the difference between the simulation outcome and the empirical data. We calibrate the model parameters by minimizing weighted mean square error (WMSE). During calibration, we penalize underestimation twice more than overestimation because field experts believe that empirical data are more likely to be underreported than overreported.

### Additional analyses.

#### L3 burden analysis.

Intuitively, when there are few L3s in a water source, EFs can have limited impact, whereas when there are many L3s, it has the potential for greater effect on total infectivity. Thus, for the best-performing EFs, we record the L3 burden in the water per month over multiple replications of the simulation, which allows us to compare the EFs in a more meaningful way.

#### R_*0*_ analysis.

We use the simulation model to estimate the basic reproductive number, often denoted by $R_{0}$, which is defined as the average number of secondary infections produced by an infected individual in an otherwise susceptible host population.^30^ We do this by parametrizing the basic reproductive number with respect to the month that the worm emerges, denoted by $R_{0}\left(m\right)$ for m=1, 2,…,12. For a given month m, $R_{0}\left(m\right)$ is computed by modeling a population of 800 dogs (approximately the largest dog population within a village with a history of GW infections) to which is introduced a single infected dog, whose worm emerges in the mth month. The outcome measure is the number of dogs that have acquired an infection by the following year. The simulation is used without interventions, and the average over 100 replications is taken as $R_{0}\left(m\right)$ for each value of m. We finally estimate $R_{0}$ as the sum of monthly $R_{0}\left(m\right)$ values weighted by the relative L3 burden per month.

## RESULTS

**Figure 5.**
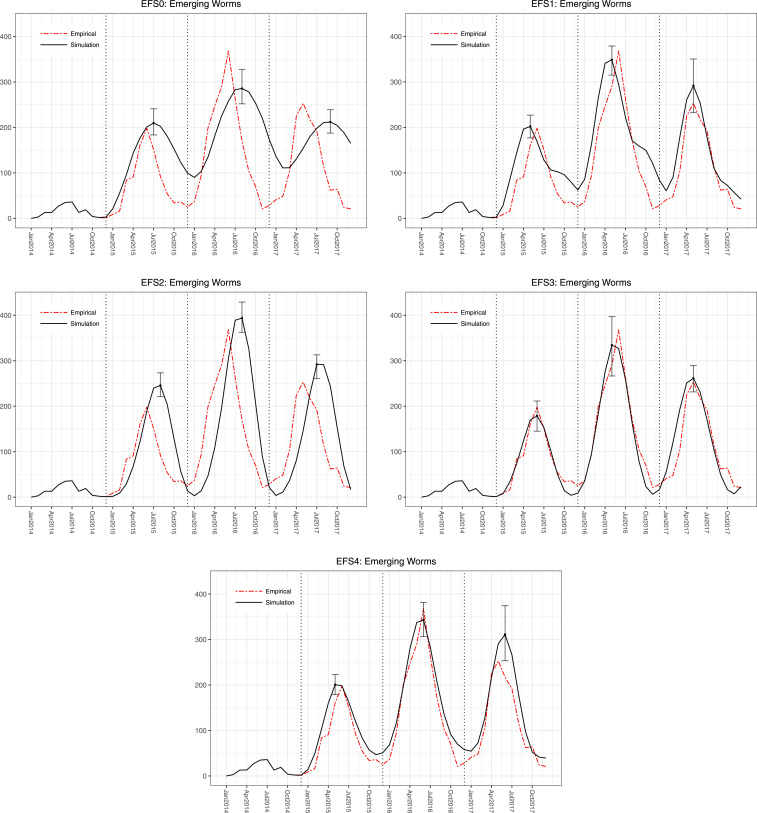
Simulated number of worms emerging from dogs for years 2014–2017, calibrated with each of the (fixed) environmental factor scenarios. Error bars indicate 95% simulated intervals at each peak. (Simulated number of dogs with emerging worms is given in Supplemental Appendix Section 3.1.) This figure appears in color at www.ajtmh.org.

The first analysis calibrates the infectivity parameters while keeping the EFs fixed to one of the five scenarios (Figure 4). The average WMSE for each scenario is summarized in the first column of Table 1. EF-S4 (combined rainfall and temperature) scenario resulted in the best fit (lowest WMSE). EF-S0 had the worst fit, with WMSE more than twice that of EF-S4. Figure 5 illustrates the fit of all five models by comparing the simulated number of worms emerging from dogs with the corresponding empirical data.§§

**Table 1:** Average WMSE is given for the calibrated simulation model with either the EF distribution fixed (second column) or calibrated (third column)

Fixed or initial scenario	Fixed EF	Calibrated EF
EF-S0 (none)	1,069.88	1,068.87
EF-S1 (temperature)	620.35*	342.45**
EF-S2 (rainfall)	1,259.65	360.95*
EF-S3 (cumulative rainfall)	538.52*	426.53
EF-S4 (temperature and rainfall)	444.68*	352.76*

EF = environmental factor; WMSE = weighted mean square error. The best-performing scenario (i.e., least WMSE) is indicated by double asterisks (**), and the next best scenarios are indicated by single asterisks (*).

**Figure 6.**
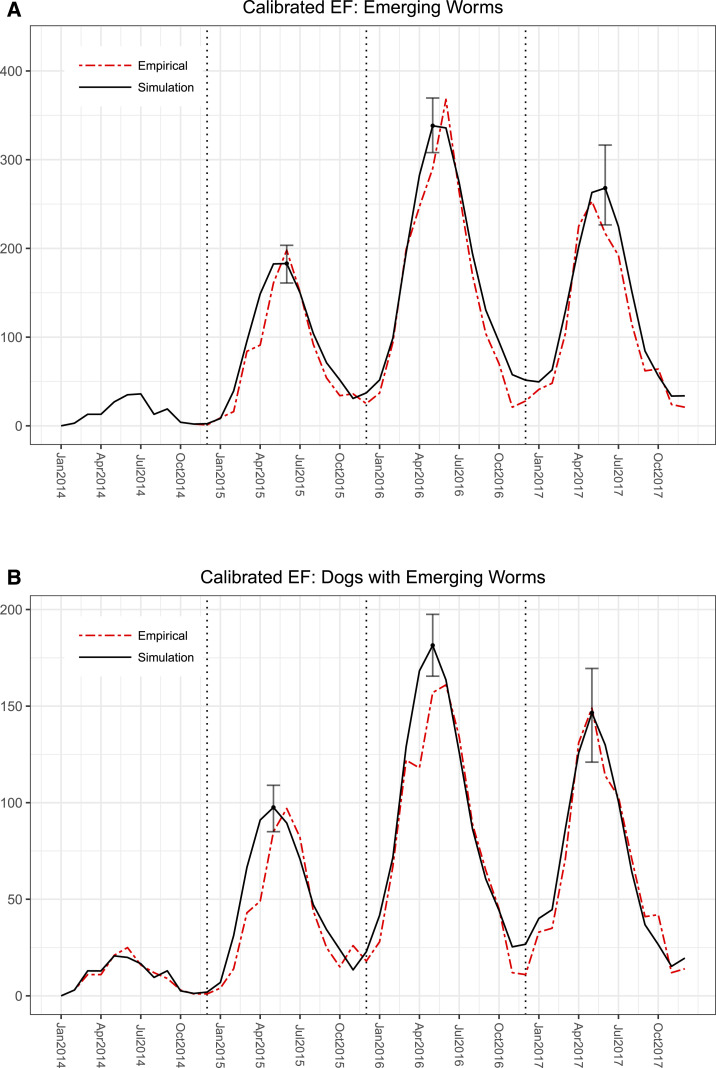
Best model when calibrating environmental factor (EF) distribution (initialized with EF-S1). The simulated number of worms emerging from dogs for 2014–2017 is shown in (**A**), and the simulated number of dogs with emerging worms is shown in (**B**). Error bars indicate 95% simulated intervals at each peak. This figure appears in color at www.ajtmh.org.

The second analysis calibrates both the infectivity parameters and the EF distribution when it is initialized to one of the five scenarios (Figure 4). The average WMSE for each scenario is summarized in the second column of Table 1. The models with the best fit according to WMSE resulted from the EF-S1 (temperature), EF-S2 (rainfall), and EF-S4 (combined) scenarios. Figure 6 shows the best fit model with respect to the number of emerging worms and the number of dogs with emergent worms. Calibrated EF distributions can be found in the Supplemental Appendix Section 3.1.

Under both analyses, EF-S0 performs substantially worse compared with all other EF scenarios. When EF scenarios are fixed, scenarios EF-S1, -S3, and -S4 perform the best, whereas after calibrating the distributions, scenarios EF-S1, -S2, and -S4 perform the best.

**Figure 7.**
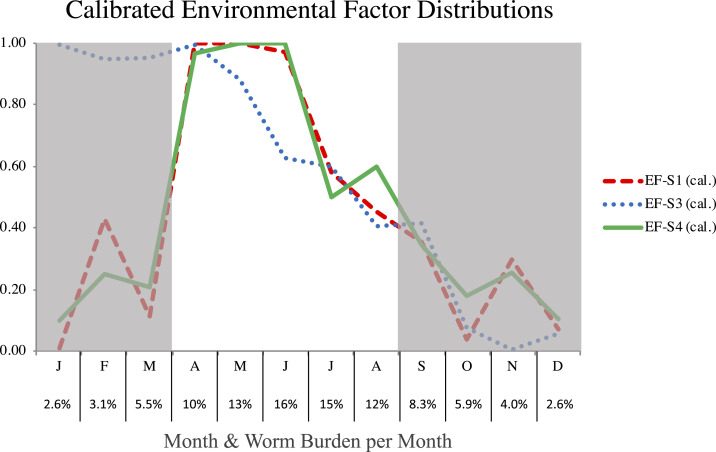
Comparing the three best-performing calibrated environmental factors (EFs). Percentages indicate the relative L3 burden in the water per month resulting from EF-S4, which is greatest from April to August, peaking in June. This figure appears in color at www.ajtmh.org.

After calibrating the EF distributions, we compare the final distributions over the period of greatest L3 burden in the water. The relative L3 burden per month is reported in Figure 7 for the calibrated EF-S1, -S3, and -S4 coefficients. Using 10% of the relative L3 burden as a threshold, we say the burden is “high” between April and August and “low” in the winter months (September through March, indicated by gray in Figure 7). The calibrated EF-S1, -S3, and -S4 coefficients are most similar from April to August, with high values in April tending to decrease until October.

**Figure 8.**
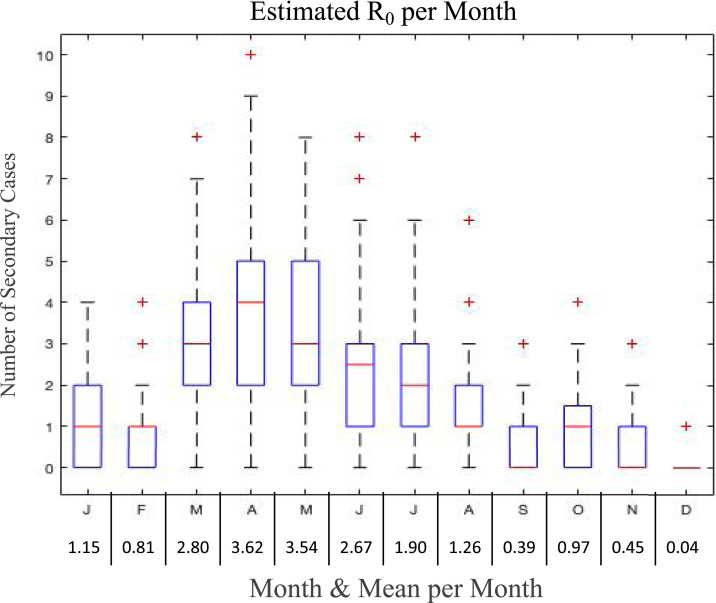
Box plots for $R_{0}\left(m\right),\;m=1,2,\ldots ,12$. Horizontal red lines indicate the median numbers of secondary cases across 100 replications, and outliers are marked by red (+). Mean values, that is, $R_{0}\left(m\right)$, are reported in the table below each month. This figure appears in color at www.ajtmh.org.

Results of the $R_{0}\left(m\right)$ analysis, when using the model with best fit parameters (including calibrated EF), are illustrated in Figure 8. As expected, the shape of the monthly $R_{0}\left(m\right)$ distribution follows the shape of the EF. It is observed that $R_{0}\left(m\right)$ is usually less than one for the winter months (*m* = 2, 9, 10, 11, and 12) and reaches a maximum of 3.6 in April; however, individual replications reached as many as 10 secondary cases in April. We estimate the basic reproductive number as the weighted average of these monthly values, weighted by monthly L3 burden, which yields $R_{0}=2.0032\approx 2$.

## DISCUSSION

Our results indicate external seasonality factors are important, in addition to the life cycle and incubation period of the pathogen. Of the hypothetical EF scenarios, the best fitting is EF-S4 (combined rainfall and temperature) followed by EF-S3 (cumulative rainfall) and EF-S1 (temperature). Through calibration of the EF distributions, three converged to a similar distribution. Furthermore, during the months with the highest L3 burden (April to August), the best-performing EFs have high infectivity in April and then steadily decrease. Possible interpretations include that these scenarios capture changes in availability of shallow pools of water with greater density of copepods or other fauna, life cycles of fauna driven by EFs, or human and dog behaviors. For example, the seasonality of human transmission in West Africa is commonly associated with agricultural, fishing, and/or migratory behaviors,^21,29^ and it may be the case that related dog behaviors during these activities also increase the risk of infection.

We present an estimate for the basic reproductive number, varying monthly, for GWs in dogs, which is about 2.0 on average, with an average of 3.6 for a worm emerging in April. The variability of the estimated *R*_0_ value across months is insightful, as many villages in Chad do, in fact, experience a wide range of secondary cases in their first year of dog infections. We note that the *R*_0_ value for dogs is not necessarily representative of the *R*_0_ value for humans because the transmission characteristics may differ. The *R*_0_ analysis provides further evidence of the most important time periods for intervention, that is, April, in particular. This can be used to target interventions based on timing or related activities.

Our simulation of GW infections, with only modest adjustments to the parameters and EF distribution, can be generalized to almost any time period or population of definitive hosts (e.g., most mammals). Currently, surveillance systems in place for mammal infections besides dogs are limited. Understanding dog and other mammal infections will be critically important for program managers, especially because the WHO certification for eradication now includes the absence of GWs in dogs.

### Limitations.

In 2018, the number of dog infections increased to the largest magnitude (in sum and at peak) seen since 2015. However, the simulation model, calibrated to the data from years 2014–2017 and with 2017 intervention levels held constant for 2018, predicted a decrease in the peak number of dog infections after 2017. In talking with public health experts, the difference is most likely due to increasing surveillance over time. There may also be some discrepancies in reported intervention levels and their real-world effectiveness.

By their nature, mathematical models and computer simulations are simplified versions of the real-world phenomenon based on a number of assumptions. For example, we used seasonal environmental data (i.e., rainfall and temperature) as a proxy for related systems (e.g., surface water) and behaviors (e.g., fishing and farming) that are not well quantified. Despite the simplifications, the simulation model captures a reasonable representation of the natural transmission pathway(s) of GWs in Chad dogs, as shown by the strong fit.

This research study also lays the groundwork for future work on the transmission of GWs. Although our sensitivity analysis on multiple water sources (see Supplemental Appendix Section 3.4) showed consistency with seasonality and the qualitative conclusions in this study, it would be useful to construct a full-scale model with multiple water sources. In addition, this study captures the interventions that were put in place in Chad, while setting a foundation to study the effect of interventions differing in type or level in future studies. Ultimately, this will allow researchers and health experts to assess what interventions can help promote the reduction or eradication of GW disease.

## Supplemental appendix sections

Supplemental materials

## Footnotes

† This refers to either *paratenic* or *transport* hosts, which may play an important role in Guinea worm transmission in Chadian dogs.^2–5^

‡ https://www.cartercenter.org/health/guinea_worm/index.html.

§ It is notable that dog behaviors are associated with human behaviors (e.g., farming and fishing), but to what degree is unclear.

** In the field, tethered dogs maintain a small risk of contracting Guinea worms because they are either not properly tethered or not provided safe water and food during this period. We examine this imperfect intervention under greater scrutiny in future work.

†† “In other villages [in Ghana], water can be obtained from perennial ponds and maximum disease transmission occurs when the water level is lowest, at the end of the dry season, and continues until the water level rises in the early wet season.” (Watts^21^)

‡‡ “As ponds shrink in a long dry season, use-rates escalate in the few remaining water sources and water contact by infective carriers (in endemic zones) produces greater rates of infection of the water fleas. In addition, as ponds shrink in volume, in the dry season, water flea densities increase commensurably… Accordingly, pond users are not only at greater risk to dracunculiasis, they also become unwitting collaborators by consuming greater volumes of water.” (Hunter^29^)

§§ The comparison between simulated number of dogs with emerging worms and the associated empirical data is included in Supplemental Appendix Section 3.1.
