# Supplementary material for: Agent-Based Simulation for Seasonal Guinea Worm Disease in Chad Dogs
Source: Am J Trop Med Hyg. 2020 Sep 8;103(5):1942–50. doi: 10.4269/ajtmh.19-0466 (PMC7646815; doi:10.4269/ajtmh.19-0466)
Supplement: Supplementary file 1 [file tpmd190466.SD1.pdf]

Computational Appendix for "An Agent-Based Simulation for Seasonal Guinea Worm Disease in Chad Dogs."

Authors: Tyler Perini*[1], Pinar Keskinocak[1], Zihao Li[1], Ernesto Ruiz-Tiben[2], Julie Swann[1], and Adam Weiss[2].

Institutions: (1) Georgia Institute of Technology, (2) The Carter Center.

1. SUPPORT FOR SIMULATION MODEL, PARAMETERS AND OPTIMIZATION.

Our simulation is designed with the following modeling assumptions. It simulates a population of 30,000 dogs, which is approximately the population of dogs in villages with infections in Chad (see Appendix Section 1.2 for details), and a single water source as the reservoir for copepods. Where appropriate, other modeling decisions, e.g., Abate treatment, are adapted with the understanding that this single water source stands in for multiple water sources. Dogs are modeled as individual agents, GWs as (partial) agents, and the water source is the channel for infections.

A single water source for transmission is a simplifying modeling assumption that is consistent with all previous mathematical models[12-16] even though transmission in Chad is likely occurring through hundreds of disjoint water sources. While a multiple water source model would provide a more accurate representation, it would greatly increase the complexity of the model (beyond the scope of this paper), and it would likely affect the volume, rather than the seasonality, of transmission, where the latter is the primary focus of this work.

With some probability, dogs acquire infection from the water source via consumption of water or (short-term) transport hosts, and dogs with patent infections (emergent worms) infect the water source. Dogs remain susceptible to new infections whether they are already infected or not, and one or more GWs emerge from an infected dog. The simulation is initialized with data from 2014 (number of infected dogs and number of emerging worms), and the remaining data from 2015-2017 are used for calibration.

1.1) Weather data. Rainfall and temperature data was collected from the National Centers for Environmental Information's Climate Data Online database [1]. From this database, we use data from all 17 weather stations in Chad that report daily precipitation and temperature

measurements[1] over the years 2013-2017. Since precipitation is reported as a single quantity over unequal time intervals (e.g., 2 reports of 6-hour time interval or 1 report of 24-hour time interval), we use the *frequency* of reported positive amounts of rainfall from all weather stations over the month as a proxy for the *amount* of precipitation per month. We note that the distributions for frequency of reported positive rainfall and sum of reported rainfall amounts (when units are ignored) are about the same, but the former is in fact more interpretable. The mean temperature for the month is taken as the reported daily mean temperatures averaged over all weather stations and over all days of the month. The resulting monthly averages are shown in Table 1.

1.2) Dog population. There are an estimated 60,000 dogs in all of the villages under surveillance (indicated by grey circles in Figure 1); however, there are many villages under surveillance that had no infections during 2014-2017. Within the villages with dog infections over this time period (illustrated by red circles in Figure 1), the estimated dog population is less than 30,000. Therefore, to keep computation time reasonable, we use 30,000 as the total dog population in the simulation.

Table 1. The computed values for representing rainfall and temperature data. Rainfall is represented by the ***frequency*** *of positive reports of rainfall per month (averaged over five years), and temperature is represented by the mean of reported daily temperatures per month (averaged over five years).*

Month	Rainfall (ρ)	Temperature (θ)
J	0.25	79.5
F	0.00	84.4
M	3.75	92.3
A	11.50	94.4
M	32.75	93.8

[1] Since precipitation is reported as a single quantity over unequal time intervals (e.g., 2 reports of 6-hour time interval or 1 report of 24-hour time interval), we use the *frequency* of reported positive amounts of rainfall from all weather stations over the month as a proxy for the *amount* of precipitation per month. The mean temperature for the month is taken as the reported daily mean temperatures averaged over all weather stations and over all days of the month.

J	47.50	90.3
J	68.00	86.1
A	87.75	83.3
S	64.50	85.0
O	23.75	88.3
N	2.25	86.6
D	0.00	80.2

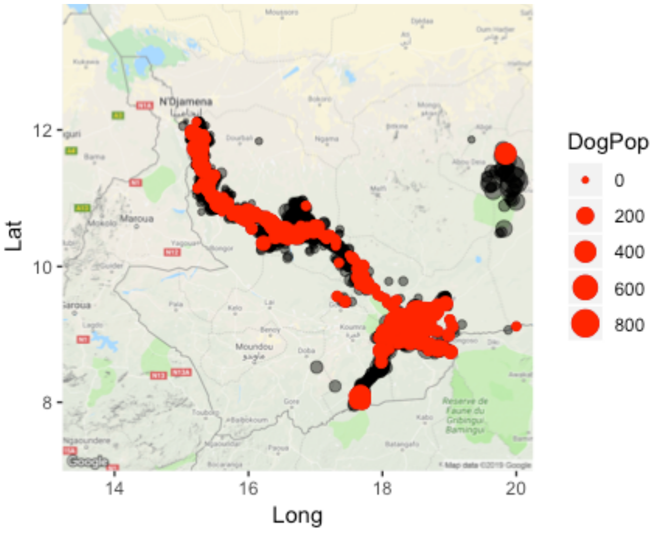

Figure 1. Villages under surveillance (gray) and villages with at least one infection (red) over the years 2014-2017 in Chad. The size of the circle represents the estimated dog population per village.

1.3) Single vs. multiple water sources. Consistent with previous mathematical models, this simulation was designed with a single water source as the channel of transmission. In reality, not all dogs in Chad have access to a shared water source, but most dogs at the village level do share at least one water source. The current model can be readily reduced in size to represent smaller populations with a single shared water source. On the other hand, dogs that have access to *multiple* water sources are at greater risk for consumption and spreading of GW larva. However, the infectivity level of each individual water source is likely less than what is represented in a single water source model since all the L3s would be distributed among separate locales. Even while seasonality should not change much, one would expect greater complexity in the infection dynamics of a model that includes multiple water sources. The current model is the first step in developing more complicated models, e.g., a multiple water source model that includes a contact network between dogs and ponds. From more advanced models that are closer to reality, new transmission dynamics may be observed which may closer match empirical data. Future work will develop such a model, in particular moving towards a decision support tool to aid the selection of interventions.

1.4) Life cycle of Guinea worm. Figure 2 summarizes the details of the GW life cycle. At the beginning of its life cycle, a GW released into a water source requires an intermediate host, a

crustacean called a copepod (*Cyclops*), where the larvae develops into its *infective* third stage (L3). The simulation accounts for copepods indirectly, by capturing the amount of time required for a GW to develop into an infective L3 larvae, i.e., 10-15 days. It is assumed that the larvae remain infective in the water source for a (fixed) 30 day duration; we discuss this further in Section 1.4. The dog (human, or other mammal) is the primary host where the parasite reproduces, and after an *incubation period* of about one year, a gravid female worm emerges from the skin of the host. The most cited range for the incubation period is 10-14 months with mean of 12 months [2-4], but we discuss this further in Section 1.5. In the simulation, we use an incubation period that is uniformly distributed between 10 and 14 months. Emerging worms can then infect any water source in which they are submerged by releasing tens of thousands of larvae. See Muller [2,]Eberhard, Ruiz-Tiben, Hopkins, Farrell, Toe, Weiss, Withers Jr, Jenks, Thiele, Cotton [5] for more details of the GW life cycle.

We now describe the simulation's representation of the GW life cycle without active interventions, which are further discussed in Section 1.4. Each infected dog is assigned a random number of worms that will emerge in the following year, where this random number comes from a discrete distribution computed from the empirical data set. Each worm is assigned an incubation period chosen (uniformly) randomly between 10 and 14 months (this period is further discussed in Section 1.6). All dogs interact with the water source once a day both for drinking and, when the dog has an emerging worm, for infecting the water. Note that we refer to the single collection of larvae released by an emerging worm at one time as a *cohort*. Since the number of larvae in a cohort is random, on the order of several thousand, it would be intractable to track each individual larva (thus, GWs are *partial* agents); instead, the simulation keeps record of the number of *cohorts of infective larvae* in the water source, which we refer to as n. Once an emergent worm has infected the water source, two timers are set: First, in 10-14 days (randomly chosen), all the larvae from this cohort become infective L3 larvae, so the simulation increments $n \leftarrow n + 1$, and thus the water source becomes *more* infective. Second, in 30 days all the larvae from this cohort will die in the water source, so the simulation decreases $n \leftarrow n - 1$, and thus the water source becomes *less* infective. A dog drinking from the infected water source is infected with some probability as a function of (1) the number of cohorts of infective larvae in the water

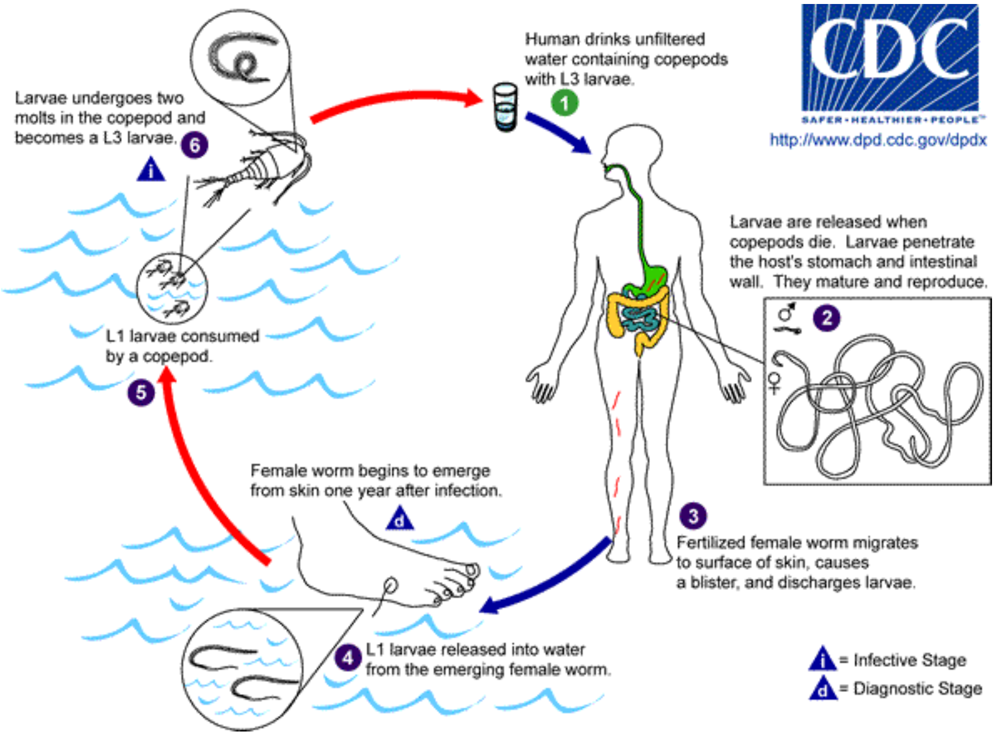

Figure 2. Diagram depicting the lifecycle of a GW, which is the same in dog hosts as it is in human hosts. Figure from the CDC.

source, n, (2) the monthly weather factor, as discussed in Section 1.7, and (3) the Abate intervention level, as discussed in Section 1.4. Further details of the infectivity function are discussed in Section 1.5. Every dog is always susceptible, i.e. a dog can be re-infected any number of times throughout a year, where each new infection accumulates an additional randomized number of worms, and we assume no dog has immunity after an infection.

We make two additional notes. First, every emerging GW has only one chance to infect the water source, and the increase in infectivity due to a new infection depends only on the current n and the shape of the infectivity function, discussed in Section 1.5. Experiments by [2] have shown that an emerging worm can actually infect a water source multiple times and that the number of larvae released decreases upon each immersion. From this, we assume that only the first exposure is the most important per worm and all worms are equally fertile. Second, when a dog becomes infected from the water source, the simulation does not decrease n, again because this represents the number of *cohorts*, each of which includes several thousand infective larvae, whereas a new infection is usually caused by the consumption of just a few larvae.

1.5) GW larvae duration. Without a copepod as a host, the larvae do not survive more than a few days. After ingestion by a copepod, larvae require 12-14 days to become infective according to most estimates, although this depends on water temperature and species of copepod [2,4]. Once a dog infects the water source with a new worm, the simulation sets a timer between 10 and 15 days (uniformly random); only once the timer has completed does the infectivity of the water increase. The infectivity of the water decreases again once the larvae dies (presumably when its copepod host dies naturally or is consumed by another predator); we refer to this time period of infectivity within the copepod host as the GW larva *duration*.

After the completion of the maturation period between first stage and infective stage, the exact duration of GW larvae in a given water source is unknown. This duration period seems to depend mostly on the intermediate host, which is difficult to estimate and depends on species and age of copepod, geographic location, climate, availability of food, predator behaviors, features of the water body (e.g., volume, temperature, etc.), among other factors [6]. There is great inconsistency amongst the published mathematical models for GW surrounding the death rate of GW larvae and/or copepods, as summarized in Table 2. Many of these modeling papers—in particular, Netshikweta, Garira [7,]Smith, Cloutier, Harrison, Desforges [8,]Link, Victor [9]—estimate or compute these parameter values without calibrating to or validating with empirical data. The two sources used for observed or measured values seem to have been cited incorrectly. First, the time to reach infective stage, i.e., 14 days as reported by Ruiz-Tiben, Hopkins [3], has been cited as the time a larva can survive once it has reached the infective stage [8]. Second, it is unclear how the laboratory results for the mortality rates of copepods, reported by Carlotti, Nival [6], were adapted into the parameter value used for the death rate of copepods [10]. Furthermore, the reported estimates came from the species *Centropages typicus,* collected from a bay in France and kept in highly controlled lab settings, so it would be difficult to translate the study's results to a natural setting for other species of copepods.

We note that our aim in highlighting these inconsistencies is not to disparage these authors or their work; rather, we intend to underscore how difficult it is to find relevant published data to suit these parameters, which are mostly unknown. In particular, the time that GW spends within its hosts (intermediate then primary) deserves more research.

Given so little information, for the simulation, we assumed the duration of GW larvae to be a fixed 30 days within a copepod. In Section Y, we analyze the sensitivity of the simulation to changes in this assumption.

1.6) Incubation period. The most commonly published range for the incubation period of GW within a host is between 10 to 14 months. Unfortunately, it is unknown exactly what the distribution of the length of the incubation period is, or even what shape it takes, or how it may differ between human and dog hosts. Table 3 summarizes the best sources we could find for this widely-used estimate for the bounds and mean of the incubation period. Most of the time, authors simply cite the Muller [2] paper's estimates. The exact distribution of the incubation period deserves more research. For now, the simulation uses an incubation period that is uniformly distributed between 10 and 14 months (average of 12 months). In Section 3.4, we analyze the sensitivity of the simulation to changes in this distribution.

Table 2. Summary of the inconsistencies in citations for the death rate of GW larvae and/or copepods.

Publication	Original text (in quotes) along with estimate (in bold)	Citation, Estimated (from model), or blank if original author.
Ghosh, Tiwari, Mandal, Martcheva, Chattopadhyay [10]	"Natural death rate of Guinea worm larvae" = **25.92 per year** [by reciprocal: lifespan ≈ 13.89 days]	Smith, Cloutier, Harrison, Desforges [8]
Smith, Cloutier, Harrison, Desforges [8]	"Parasite death rate" = **26 per year** [by reciprocal: lifespan ≈ 13.85 days]	Ruiz-Tiben, Hopkins [3]
Ruiz-Tiben, Hopkins [3]	"These larvae (first-stage) must then be ingested whole by certain species of water copepods, invade the copepod body cavity and become infective (third-stage) after 14 days of development." [Note that 14 days is the time required to *reach* the third stage, not the length of time that the larvae survive after reaching this stage.]	
Ghosh, Tiwari, Mandal, Martcheva, Chattopadhyay [10]	"Death rate of copepods" = **1.44 per year** ≈ 0.004 per day [by reciprocal: lifespan ≈ 250 days]	Carlotti, Nival [6]
Carlotti, Nival [6]	"A minimum mortality rate (0.01 to 0.02 [per day]) was present before the mode of the duration distribution. Beyond the mode, mortality increased to a maximum of 0.1 [per day] for those individuals which… [Summarized from Table 1: on average, it took about 14 days for a copepod to mature from CI to end of CV stage. Summarized from Table 2: Other authors using different methods often found an average of 30 days between egg to adult.]… [T]he intrinsic mortality rate increased[, starting] at a value of 0.02 [per quarter day = 0.08 per day], it reached a maximum of 0.1 [per quarter day = 0.4 per day]… We can estimate graphically the mean of this maximum death rate at around 0.1 to 0.12 [per quarter day = 0.4 to 0.48 per day]. The natural mortality rate of the copepod… varies from 0.01 to 0.1 [per day]."	

Netshikweta, Garira [7]	"Natural decay rate of copepods" = **0.005 per day**	Link, Victor [9]
Link, Victor [9]	"Natural death rate of larvae = **2.5 per time"** (in disease-free equilibrium) or "**0.25 per time"** (in endemic equilibrium)<br>"Natural death rate of copepods = **1 per time"** (in both disease-free and endemic equilibria)	Estimated
Netshikweta, Garira [7]	"Disease induced death rate of copepods" = **9e-6 per day**	Estimated

Table 3. Summary of sources and limited details for the incubation period of GW within its host. Number of citations are estimated from Google Scholar.

Muller [2]<br>[179 citations]	• "The uterus of the female worm at 8-9 months is filled with developing eggs… The embryos break free of the egg shell and are usually fully formed by 10 months."<br>• "The mature female emerges 10-14 months after infection. In man this information is based chiefly on the accounts of travelers making a short visit to an endemic region, includes one experimental infection and is strongly supported by epidemiological evidence… A similar development period to that in man has been observed in laboratory infections in dogs, and in nine rhesus monkeys infected once only I found that the first worms emerged 321-360 (mean 340) days later [10.7-12.0 (mean 11.3) months later]." [In-text citations omitted for readability.]
Cairncross, Muller, Zagaria [4]<br>[132 citations]	• "The females move down the muscle planes and by 10 months have grown greatly, with the uterus being filled with larvae. They emerge about 1 year after infection… The process from ingestion of a larva to emergence of an adult worm typically takes about a year, enabling transmission to occur annually at the most favorable season. However, in individual cases it can last from 10 to 14 months."
Ruiz-Tiben, Hopkins [3]<br>[105 citations]	• "Infected people do not experience symptoms until 10–14 months later when the gravid adult female worm(s)… extrude[s] to become exposed to the environment outside the human body."

1.7) Intervention methods. The simulation incorporates three interventions.

(i) Tethering: Dogs are kept on a leash for 30 days after the emergence of a worm to prevent the dog from infecting any water sources. In the simulation model, an input parameter controls the tethering coverage per year. Once a dog reaches its emergence date, with some probability (given by that year's coverage) that dog is tethered for 30 days, thus preventing it from infecting the water source *and* preventing the dog from drinking from the water source during that timeframe. The tethering compliance rates reported by the Carter Center for years 2014-2017 are 40%, 68%, 63%, and 76%, respectively, [11]; these values are used in the simulation as the tethering coverage probabilities.

(ii) Abate treatment: a larvicide called Abate (a.k.a., *temephos*), which paralyzes and eventually kills copepods in the water for up to 30 days, is used to reduce the water source's infectivity. Abate water treatments have a two-pronged implemented in the simulation. First, Abate *reduces* the number of infective GW larvae in the water source; however, since there is only one water source in our model, instead of eliminating all larvae in the water, the simulation only eliminates a proportion of them. This is a reasonable assumption because, in practice, it is difficult to reach 100% effectiveness when applying Abate because of water bodies that are too large to treat at once, too difficult to measure volume accurately, or too turbid. In the simulation, the reduction of GW larvae due to Abate occurs once every 30 days during a year when Abate coverage is positive. Abate's second effect is it *prevents* a proportion of the dogs from getting infected. Since our simulation uses one water source as a *proxy* for multiple water sources, we prevent a proportion of the dogs from interacting with the infected water source each day. Both proportions (of larvae eliminated in the water source and of dogs prevented from drinking infected water) are determined by the year's Abate coverage parameter. The Carter Center reports that number of dogs in villages treated with Abate was 0% for 2014-2015 and 40% for 2016-2017 [11], which are the values used in the simulation.

(iii) Other interventions: this generic category represents the various efforts to prevent dogs from interacting with the infected water source, including public education efforts, distribution of water filters and pumps, and burying fish entrails during food preparation (instead of allowing dogs to eat entrails). To capture these "other" interventions per year, the simulation has a parameter indicating the proportion of dogs that are prevented from new infections per year. The effect of this parameter is similar, and in addition to, the preventative effect of the

Abate treatment. For example, if there is a 40% coverage of the Abate treatment and 30% coverage of "other" interventions, then a total of 70% of dogs are protected from infection in the simulation. This parameter is calibrated in the parameter calibration procedure.

1.8) Probability of infection. Unlike the transmission probabilities of diseases like HIV, which have been estimated over many studies [12], experimental results for the transmission probability of GW larvae through consumption of water (or any transport host) has not been published, to our knowledge. However, since it is impossible for one dog to directly infect another dog, and a dog already infected is still equally susceptible to another infection (no studies have shown either increased or decreased susceptibility once infected), then we can assume the probability of transmission is independent of the population (and density) of the dogs and each dog's individual infection history. Furthermore, the probability of dogs becoming infected with GWs via drinking water is uncertain, but deemed low, given the way dogs thump water with curled tongue while drinking and the known avoidance behavior of copepods (rapid darting away) in response to a source of disturbance of water.

To be precise, the probability of a single dog having an emerging, gravid female GW depends on several events, including: the dog consuming at least two matured GW L3 larvae via copepods (or some transport host), one of each sex; two parent L3s successfully penetrating the stomach wall of the definitive host; and both L3s surviving long enough to mate. Rather than attempt to approximate the probabilities of each of these individual events, we instead model the overall probability of the final event (a dog having an emergent gravid female worm) as a function of the population of matured, i.e. infective, GW larvae cohorts in the water source.

We propose a sigmoidal function to represent the probability of a dog getting infected during each day's interaction with the water source, which is consistent with theoretical studies of vector-borne parasites [29] and macroparasites [30] as well as experimental results on malaria parasites [31]. (The input data and parameters used in this section are summarized in Table 4: lower-case letters represent input data, and capital letters represent parameters which will be fitted.) The proposed function is S-shaped, monotonically increasing, and has a lower asymptote, upper asymptote, and inflection point. Note that since this function is representing a probability, it is necessary to have upper and lower asymptotes to bound the function between zero and one. The basic sigmoidal function, f, is of the form $f(n) = \frac{1}{1+e^{-C(n-D)}}$, where D is the inflection point, and C is a parameter controlling the curvature of the function (see Figure 3). Here, n is the

number of infective copepods in the water, which we call the *worm burden*. Let $F_L \geq 0$ be the minimum rate of infection and $F_U \leq 1$ be the maximum rate. We define the *basic infectivity function* as a function of the worm burden, *n*:

$$F(n) = [f(n) - f(0)](F_U - F_L) + F_L.$$

Note that depending on the values of the parameters, the basic infectivity function $\mathrm{F}(n)$ could attain a shape that is nearly constant (when $F_L \approx F_U$), nearly linear (when the curvature a is close to zero), nearly exponential (when the inflection point C is very large), or "truly" s-shaped (Figure 3). These parameters will be calibrated according to the process described in Section 2 to best approximate the empirical results.

Let time be represented both by year $y = 1,2,3,4$ and month $m = 1,2,\ldots,12$. For year y, let the Abate intervention coverage be represented by $a_y \in [0,1]$, and let the other intervention coverage, which is parametrized, be represented by $B_y \in [0,1]$, where $a_y + B_y \leq 1$ for all y. We refer to the seven variables $C, D, F_L, F_U,$ and $B_{2014}, \ldots, B_{2017}$ as the *infectivity parameters*.

For month m, let the *environmental factor (EF),* $w_m \in [0,1]$, represent how the environment may influence infectivity which, for now, we treat as input data; later, we will treat it as a parameter to be fitted and refer to it as W_m to be consistent with notation. The final infectivity function is defined as a function of the worm burden, *n*, and the time in month and year, *m* and *y,* respectively:

$$F_{\text{inf}}(n, m, y) = w_m \times (1 - a_y - B_y) \times F(n).$$

The tethering intervention coverage per year y, represented by $t_y \in [0,1]$, does not appear in the infectivity function. Abate and tethering coverages are fixed values from reported data (as indicated by lowercase letters), whereas "other interventions" is a parameter that is unknown and therefore calibrated.

Note that not having a seasonal (monthly) influence on infectivity would implicitly assume that the water source is fixed in volume all throughout the year, which we know to be false in Chad which has extreme rainy and dry seasons. Consider the example in Figure 4, where there is no seasonal influence on infectivity. Even though there are about the right number of infections in the summer months, there are clearly far too many infections in the months in between summers, as well. This motivated the need for a seasonal factor that *dampens* infectivity during certain months.

Table 4. Variables used: lower-case letters represent input data, and capital letters represent parameters which will be fitted. The infectivity parameters are highlighted in grey.

n	Number of infective copepods in the water
C	Curvature of the infectivity function
D	Inflection point of the infectivity function
F_L	Minimum rate of infection
F_U	Maximum rate of infection
B_y	Other intervention coverage per year y
a_y	Abate intervention coverage per year y
t_y	Tethering intervention coverage per year y
w_m	Environmental factor per month m
W_m	Environmental factor parameter per month m

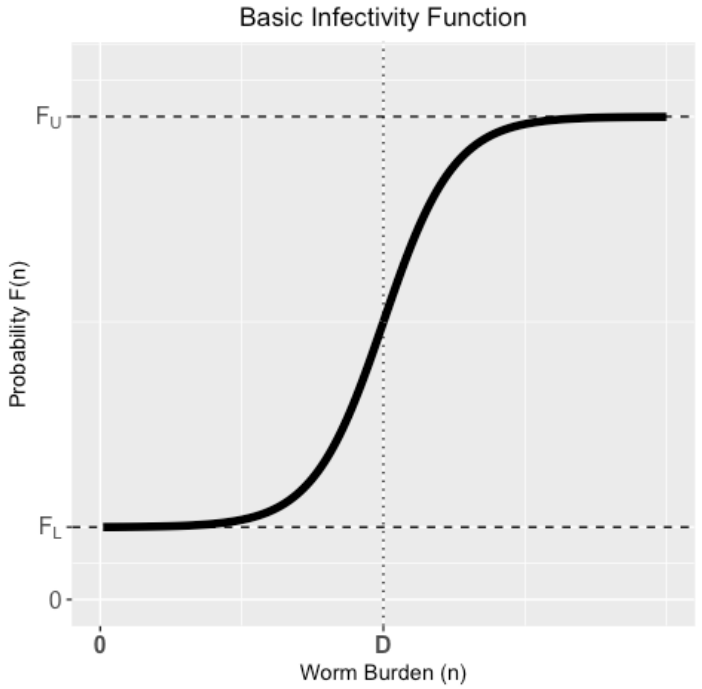

Figure 3. The sigmoidal shape of the basic infectivity function, F(n), as a function of L3 burden, n, is shown with the parameters F_U, F_L, and D. Parameter C (not shown) controls the curvature.

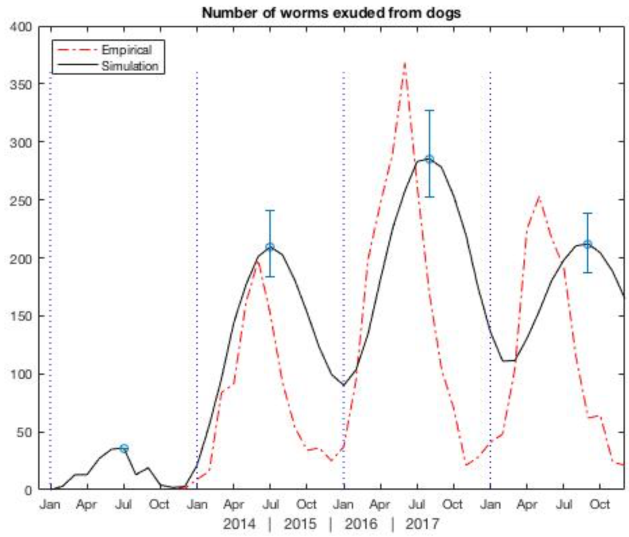

Figure 4. An example of the simulation results when there is no seasonal factor, i.e., $w_m = 1$ *for all months* $m = 1,2,\ldots,12$*. The empirical number of worms exuded from dogs is the dashed, red line. The simulation results, in solid black, is after calibrating the infectivity parameters.*

1.9) Factors driving seasonality. Muller [2], Cairncross, Muller, Zagaria [4] have observed seasonality in GW infections, which is explained by an incubation period with mean around 12 months. The timing of the season of highest infections depends on the region, e.g., the rainy season in the Sahelian zone of Africa and the dry season in the southern parts of Africa. The timing may be influenced by different climatic factors and/or human behaviors (such as agriculture), as discussed in Cairncross, Muller, Zagaria [4].

We propose the following potential EF scenarios specifically for Chad starting in 2013; it should be noted that these EF scenarios may not equally apply to another geographic region or time period. The following five hypothetical EF distribution scenarios are enumerated in Table 5.

- **EF-S0: Weather has no effect on infectivity**. For this hypothesis, $w_m = 1$ for all $m = 1,2,\ldots,12$.
- **EF-S1: Infectivity increases as temperature increases**. Using the temperature data measured by the weather stations, we set the environmental factor w_m such that it ranges from 0 to 1 proportionally with average monthly temperature, i.e., $w_1 = 0$ for January, and $w_4 = 1$ for April.

- **EF-S2: Infectivity increases as rainfall increases**. Using the rainfall data measured by the weather stations, we set the environmental factor w_m such that it ranges from 0 to 1 proportionally with average monthly rainfall, i.e., $w_2 = 0$ for February, and $w_8 = 1$ for August.
- **EF-S3: Infectivity decreases as annual rainfall accumulates**. Using the rainfall data measured by the weather stations, we set w_m such that it is highest in January, i.e., $w_1 \approx 1$, and monotonically decreases proportionally with monthly rainfall until it reaches zero in December, i.e., $w_{12} = 0$.
- **EF-S4: Infectivity is greatest before the rainy season and when temperature is high**. First, let ρ_m be the rainfall per month m and θ_m be the temperature per month m (both given in Table 1). Now we first define for all $m = 1,2,\dots,12$ (where modulo is assumed for indexing purposes):

$$w_m = \left(1 + \left(\frac{\theta_{m-1}}{100}\right)^5\right) \times \left(\frac{\rho_{m+1} + 2\rho_{m+2} + 2\rho_{m+3} + \rho_{m+4}}{3 \times 100}\right).$$

Note that this formulation takes into account the previous month's temperature as well as the next four months' rainfall. Now the final EF-S4 distribution is given by scaling w_m to range between 0.1 and 1.0, i.e., $w_m^* = \left(\frac{0.9}{\max_m w_m}\right) w_m + 0.1$.

Table 5. Five hypothetical Environmental Factor distributions to be tested.

	**J**	**F**	**M**	**A**	**M**	**J**	**J**	**A**	**S**	**O**	**N**	**D**
**EF-S0**	1	1	1	1	1	1	1	1	1	1	1	1
**EF-S1**	0	0.3248	0.8573	1	0.9585	0.7247	0.4407	0.2539	0.3657	0.5861	0.4756	0.0482
**EF-S2**	0.0028	0	0.0427	0.1311	0.3732	0.5413	0.7749	1	0.7350	0.2707	0.0256	0
**EF-S3**	0.9993	0.9993	0.9883	0.9547	0.8589	0.7200	0.5212	0.2646	0.076	0.0066	0	0
**EF-S4**	0.2023	0.3235	0.5156	0.8125	1.0000	0.9304	0.6179	0.3082	0.1485	0.1048	0.1079	0.1347

2. PARAMETER CALIBRATION PROCEDURES.

There are two sets of parameters: the seven infectivity parameters (C, D, F_L, F_U, and B_y for $y = 2014, \dots, 2016$) and the twelve EF parameters (one per month of the year). We used two approaches for calibration: infectivity parameter calibration procedure (IPCP) and alternating parameter calibration procedure (APCP). First, IPCP is used to compare hypothetical EFs by fixing w_m to one of the distributions EF-S0, …, EF-S4 and then calibrating only the infectivity parameters. Then, APCP is used to parametrize the EF, i.e. replacing w_m with W_m, such that the EF, itself, can also be calibrated; the procedure initializes the EF parameters to be equal to one of the hypothetical EF distributions, then it alternates between calibrating the infectivity parameters and the EF parameters until some convergence is reached. For both procedures, we use the following techniques.

2.1) Weighted Mean Squared Error. For calibrating the simulation, we equally consider two metrics. First, the weighted mean squared error (WMSE) between the simulated *number of worms* exuded per month and the associated empirical data. Second, the WMSE between the simulated *number of dogs* exuding worms per month and the associated empirical data. We effectively double the cost of the simulation *underestimating* the number of infections with the following formula. Let x_t be the empirical number of worms exuded per month t, y_t be the empirical number of dogs exuding worms per month t, and $\widehat{x_t}$, $\widehat{y_t}$ be the simulated approximations of those respective values. Then we define

$$WMSE(\hat{x}, \hat{y}) = \sqrt{\sum_t ([\widehat{x_t} - x_t]_+)^2 + 4([x_t - \widehat{x_t}]_+)^2} + \sqrt{\sum_t ([\widehat{y_t} - y_t]_+)^2 + 4([y_t - \widehat{y_t}]_+)^2}$$

as the function to be minimized through parameter calibration.

2.2) Latin Hypercube (LH) sampling generates samples (assignments of values to parameters) by choosing parameter values but in a random but structured manner [16]. Each parameter can take a value at ten possible levels (Table 6). The only criterion that parameters must satisfy is that the lower bound for infectivity is no greater than the upper bound, i.e. $F_L \leq F_U$. Therefore, samples that do not satisfy this criterion are eliminated, and candidates are generated until there are 100 feasible samples. Every sample's performance is measured by the average WMSE after 10 replications.

Table 6. Ten levels used for each of the infectivity parameters during Latin Hypercube Sampling.

C	0.01	0.02	0.03	0.04	0.05	0.06	0.07	0.08	0.09	0.10
D	10	25	50	75	100	200	400	500	750	1000
F_L	5e-5	8e-5	1e-4	2e-4	3e-4	4e-4	5e-4	6e-4	8e-4	1e-3
F_U	5e-4	6e-4	8e-4	1e-3	1.25e-3	1.5e-3	1.75e-3	2e-3	3e-3	4e-3
B_{2014}	0	0.05	0.1	0.2	0.25	0.3	0.35	0.4	0.45	0.5
B_{2015}	0	0.05	0.1	0.2	0.25	0.3	0.35	0.4	0.45	0.5
B_{2016}	0	0.1	0.2	0.3	0.35	0.4	0.45	0.5	0.55	0.6

2.3) Rinott's Procedure (RP) is a two-stage statistical procedure used when selecting the best system (or subset) out of a set of stochastic systems given some "indifference zone", with some probability or confidence level [17,18]. In this case, we are indifferent to a difference of less than $\delta = 10$ in the average WMSE, and we desire 95% probability of choosing the best set of parameters, i.e., $\alpha = 0.05$. First, RP uses $n = 10$ initial replications of each system to determine the standard deviation of performance. Then, with the Rinott's constant—4.29, found in the table in [19] using n, δ, α—and the sample standard deviation, the procedure computes the required number of replications in order to be *confident* in the average performance. After running the computed number of replications, RP simply chooses the parameter set with the lowest average WMSE. Because running the simulation tens of thousands of times is very costly, we limit the number of replications to 5,000; however, requiring more than this number of replications was rarely observed in practice. Even with fewer than 5,000 replications, it can take up to 10 hours for RP to compare ten systems. RP is commonly used in simulation for parameter ranking selection [20,21].

2.4) Nelder Mead (NM) search is a "direct search" (i.e., derivative-free) optimization algorithm originally designed for finding locally optimal solutions to deterministic, unconstrained problems [22]. NM search has also been used for parameter estimation/calibration for simulations, e.g., [23,24]. Matlab has encoded the NM search algorithm as the function *fminsearch*. With some simple transformations, it is possible to solve problems with upper and lower constraints on the input variables, e.g. probabilities between 0 and 1. An implementation of NM search with bounds, called *fminsearchbnd*, is freely available at Mathworks File Exchange website[2]. The objective function we use during NM search is the average WMSE after 10 replications for each set of parameter values. NM search is stopped after 100 iterations (usually most improvement in the objective occurs in the first 50 iterations)[3]. Each NM search requires on average 3 hours to complete.

2.5) Infectivity Parameter Calibration Procedure (IPCP). This calibration procedure has three steps. Throughout IPCP, the EF parameters are fixed to the distribution given by the EF scenario.

---

[2] https://www.mathworks.com/matlabcentral/fileexchange/8277-fminsearchbnd-fminsearchcon

[3] The default stopping conditions for NM *fminsearch* in Matlab includes both a relative tolerance for the input values *and* the function value; however, with stochastic function values, the relative tolerance for the latter is never satisfied.

(i). First, we use LH sampling to find a “good” set of infectivity parameter values to initialize the NM search. With 100 samples from LH sampling, the average WMSE is taken over ten replications per set. From the nine samples with the lowest average WMSE, use RP as a refining step to determine the best set.

(ii). Initialize NM search with the best values for the infectivity parameters, found from first step. NM computes 100 iterations in search for a locally optimal set of values.

(iii). Use RP again to confirm that the final set of parameter values from completion of second step performs better than all nine sets of parameter values from first step (which it almost always does).

2.6) Alternating Parameter Calibration (APCP).

This calibration procedure has four steps, which are similar to the steps in IPCP, however it includes alternating between steps (ii) and (iii).

(i). First, we fix the EF parameters to the intial EF distribution and use sampling to find a “good” set of infectivity parameters to initialize the NM search. With 100 samples from LH sampling, the average WMSE is taken over ten replications per set. From the nine samples with the lowest average WMSE, use RP to determine the best set.

(ii). Begin the alternating calibration by searching for a local optimum with respect to the *infectivity parameters*. With the EF parameters *fixed*, the current set of infectivity parameters (e.g., from the first step) is taken to initialize NM search. After 100 iterations of NM search, update the infectivity parameters with the improved values. If the relative difference in these values is less than 0.001, then the system has converged so go to step (iv). Otherwise, continue to step (iii).

(iii). Continue the alternating calibration by searching for a local optimum with respect to the *EF parameters*. With the infectivity parameters *fixed*, the current set of EF parameters (e.g., from the initial EF distribution) is taken to initialize NM search. After 100 iterations of NM search, update the EF parameters with the improved values. If the relative difference in these values is less than 0.001, then the system has converged so go to step (iv). If this concluded the maximum 20th use of NM search, then also go to step (iv). Otherwise, return to step (ii).

(iv). Each iteration of the APCP produces, in theory, a sequence of improving solutions. However, this is with respect to only ten realizations of the simulation at a time. As a final step, we use RP to compare the set of most recent combinations of infectivity and EF parameters (at

most 10) and thus we choose the best set of parameters with statistical confidence in the selection.

3. RESULTS.

3.1) IPCP and APCP.

We first summarize the results of IPCP and APCP in Tables 7 and 8 before presenting more detailed results from the calibration procedures.

Table 7. Calibrated infectivity coefficients resulting from IPCP for each EF scenario. The rightmost column indicates the average WMSE. The best performing scenario is indicated by double asterisks (**), i.e. EF-S4 highlighted in grey, and the next best scenarios are indicated by single asterisks (*).

**Scenario**	F_L	F_U	D	C	*otherInt 2014*	*otherInt 2015*	*otherInt 2016*	**Average WMSE**
**EF-S0 (None)**	7.056e-05	1.999e-03	40.28	0.0115	0.1426	0.4777	0.2896	1069.88
**EF-S1 (Temp)**	2.792e-04	1.733e-03	92.25	0.0739	0.4778	0.2135	0.2387	* 620.35
**EF-S2 (Rain)**	4.000e-04	6.000e-04	400.00	0.0100	0.5000	0.2000	0.0000	1259.65
**EF-S3 (Acc. Rain)**	6.934e-06	1.045e-03	10.05	0.0741	0.3145	0.4573	0.2990	* 538.52
**EF-S4 (Temp & Rain)**	2.904e-04	1.722e-03	94.09	0.0766	0.5163	0.2413	0.1994	** 444.68

Table 8. Calibrated infectivity parameters resulting from APCP for each EF scenario. The rightmost column indicates the average WMSE. The best performing scenario is indicated by double asterisks (**), i.e. EF-S1 highlighted in grey, and the next best scenarios are indicated by single asterisks (*).

**Initial Scenario**	F_L	F_U	D	C	*otherInt 2014*	*otherInt 2015*	*otherInt 2016*	**Average WMSE**
**EF-S0 (None)**	5.792e-05	1.997e-03	25.62	0.0125	0.1844	0.4881	0.3050	1068.87
**EF-S1 (Temp)**	3.111e-04	1.699e-03	101.43	0.0811	0.5720	0.2341	0.1726	** 342.45
**EF-S2 (Rain)**	4.073e-04	3.036e-02	824.51	0.0292	0.5984	0.2230	0.0266	* 360.95
**EF-S3 (Acc. Rain)**	5.243e-06	1.162e-03	13.16	0.0735	0.3520	0.5312	0.3428	426.53
**EF-S4 (Temp & Rain)**	2.972e-04	1.992e-03	108.44	0.0791	0.5803	0.1940	0.1234	* 352.76

EF-S0: Weather has no effect on infectivity. The table details the parameter optimization process for the EF-S0 model. *LHC* indicates the best sample from Latin Hypercube sampling, *IPCP* indicates the results from the infectivity parameter calibration procedure (EF parameters fixed), and *APCP* indicates the results from alternating parameter calibration procedure. The rightmost columns indicate the number of replications required for RP and the resulting average WMSE. The remaining figures depict the fit for the IPCP and APCP parameters, as well as the change in EF distribution throughout APCP before convergence.

**Source**	*minRate*	*maxRate*	*inflection point*	*para*	*otherInt 2014*	*otherInt 2015*	*otherInt 2016*	**Reps for RP**	**Average WMSE from RP**
APCP	5.792e-05	1.997e-03	25.62	0.0125	0.1844	0.4881	0.3050	2079	1068.87
IPCP	7.056e-05	1.999e-03	40.28	0.0115	0.1426	0.4777	0.2896	599	1069.88
LHC	8.000e-05	2.000e-03	25	0.0100	0.1000	0.4500	0.4000	1024	1185.98

<table>
<tr><td colspan="2">Results after IPCP (100 replications)</td></tr>
<tr><td>Number of worms exuded from dogs
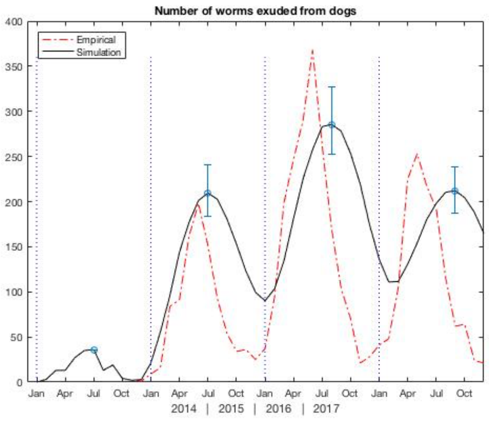
</td><td>Number of dogs exuding worms
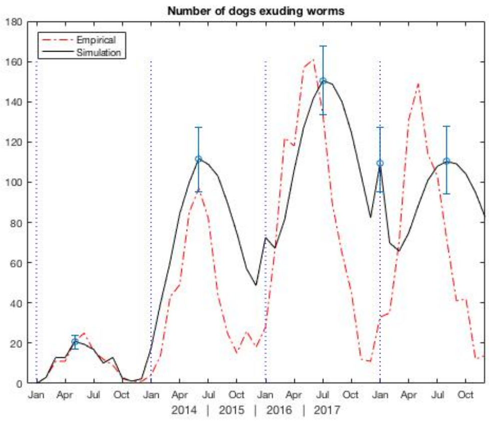
</td></tr>
<tr><td colspan="2">Results after APCP (100 replications)</td></tr>
<tr><td>Number of worms exuded from dogs</td><td>Number of dogs exuding worms</td></tr>
</table>

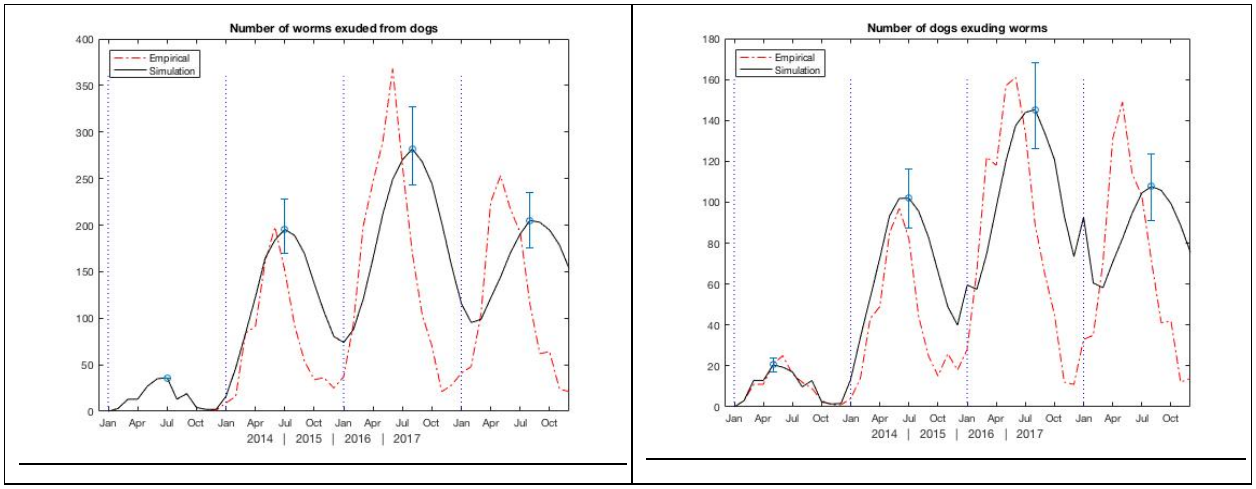

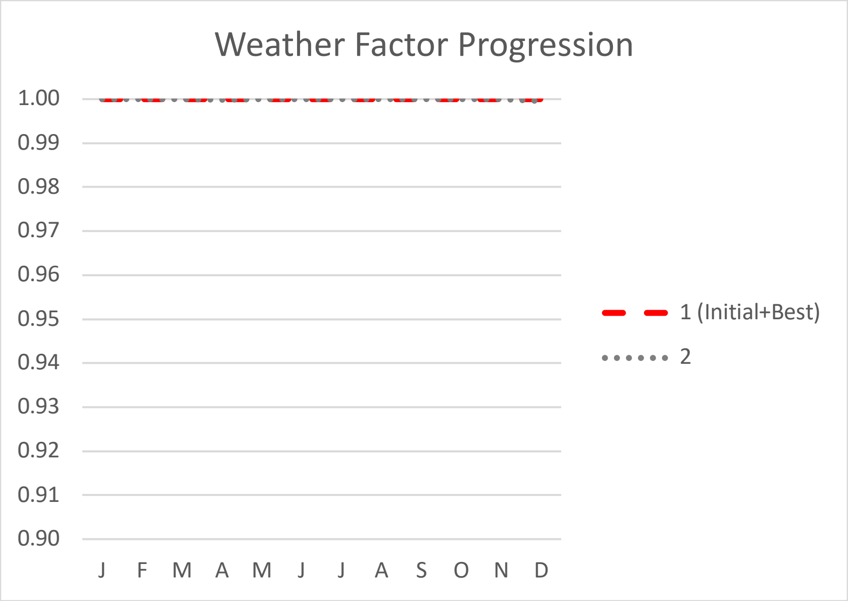

EF-S1: Infectivity increases as temperature increases. The table details the parameter optimization process for the EF-S1 model. *LHC* indicates the best sample from Latin Hypercube sampling, *IPCP* indicates the results from the infectivity parameter calibration procedure (EF parameters fixed), and *APCP* indicates the results from alternating parameter calibration procedure. The rightmost columns indicate the number of replications required for RP and the resulting average WMSE. The remaining figures depict the fit for the IPCP and APCP parameters, as well as the change in EF distribution throughout APCP before convergence.

**Source**	*minRate*	*maxRate*	*inflection point*	*para*	*otherInt 2014*	*otherInt 2015*	*otherInt 2016*	**Reps for RP**	**Average WMSE from RP**
APCP	3.111e-04	1.699e-03	101.43	0.0811	0.5720	0.2341	0.1726	348	342.45
IPCP	2.792e-04	1.733e-03	92.25	0.0739	0.4778	0.2135	0.2387	360	620.35
LHC	3.000e-04	1.750e-03	100	0.0800	0.4000	0.2000	0.2000	479	705.26

Results after IPCP (100 replications)

Number of worms exuded from dogs

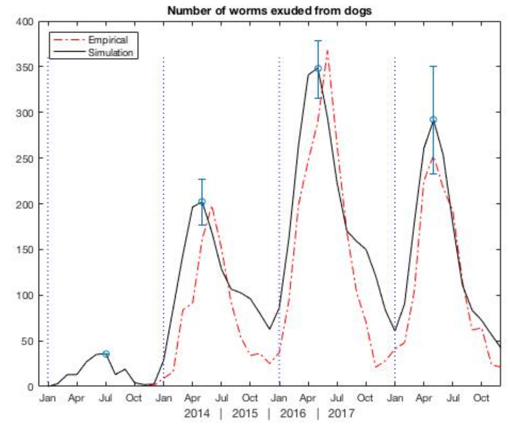

Number of dogs exuding worms

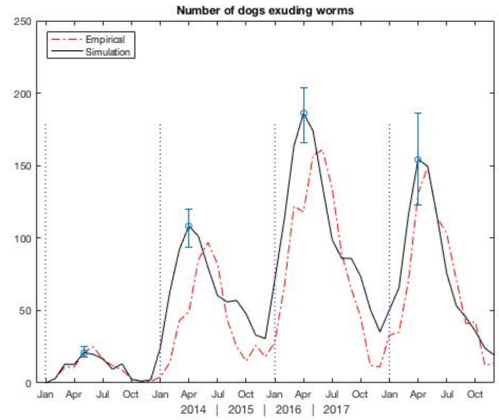

Results after APCP (100 replications)

Number of worms exuded from dogs

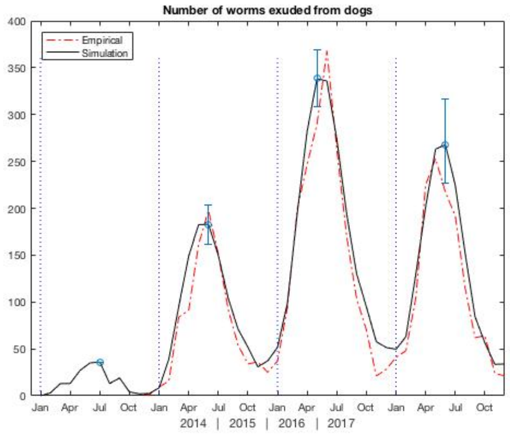

Number of dogs exuding worms

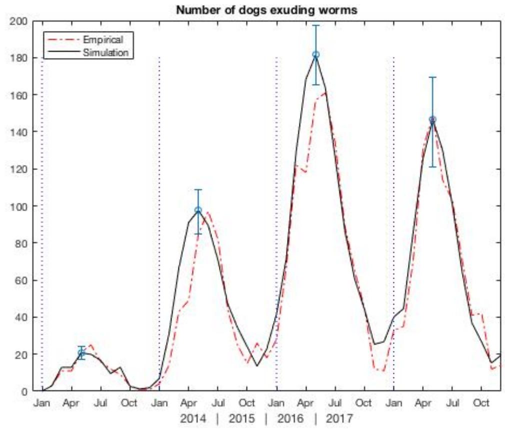

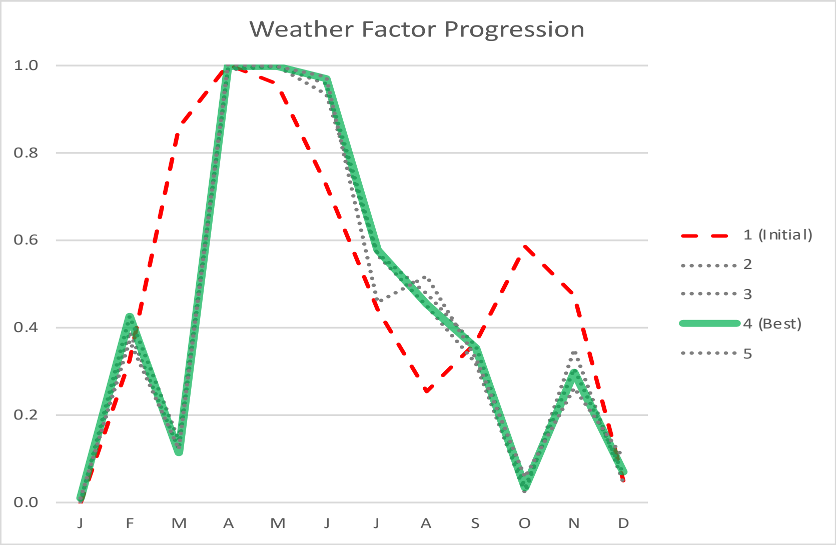

EF-S2: Infectivity increases as rainfall increases. The table details the parameter optimization process for the EF-S2 model. *LHC* indicates the best sample from Latin Hypercube sampling, *IPCP* indicates the results from the infectivity parameter calibration procedure (EF parameters fixed), and *APCP* indicates the results from alternating parameter calibration procedure. The rightmost columns indicate the number of replications required for RP and the resulting average WMSE. The remaining figures depict the fit for the IPCP and APCP parameters, as well as the change in EF distribution throughout APCP before convergence.

**Source**	*minRate*	*maxRate*	*inflection point*	*para*	*otherInt 2014*	*otherInt 2015*	*otherInt 2016*	**Reps for RP**	**Average WMSE from RP**
APCP	4.073e-04	3.036e-02	824.51	0.0292	0.5984	0.2230	0.0266	170	360.95
IPCP	4.006e-04	6.000e-04	398.51	0.0100	0.5013	0.2012	0.0009	292	1263.54
LHC	4.000e-04	6.000e-04	400	0.0100	0.5000	0.2000	0.0000	277	1259.65

Results after IPCP (100 replications)	
Number of worms exuded from dogs	Number of dogs exuding worms

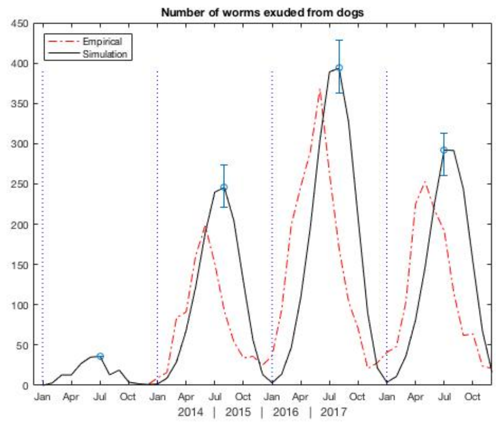	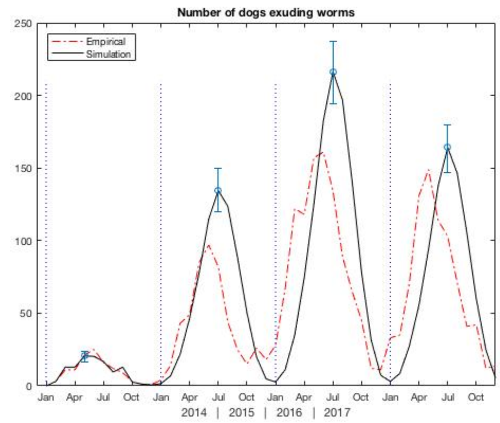
Results after APCP (100 replications)	
Number of worms exuded from dogs	Number of dogs exuding worms
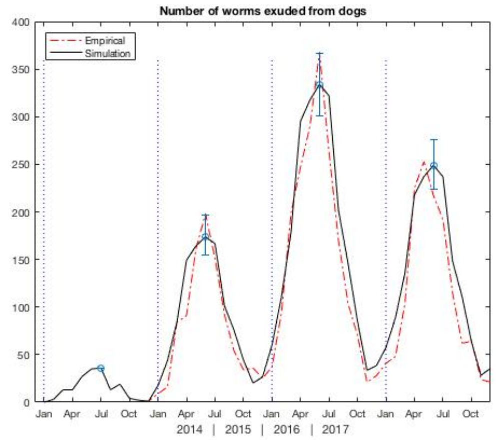	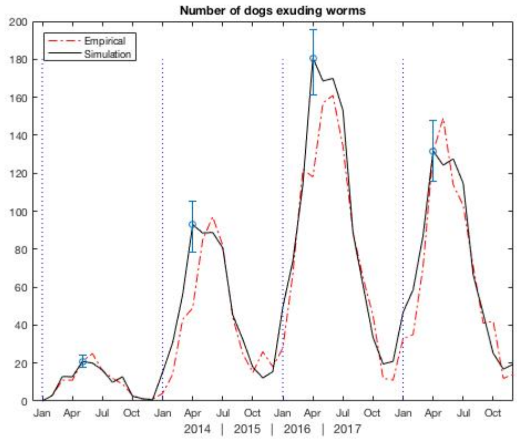

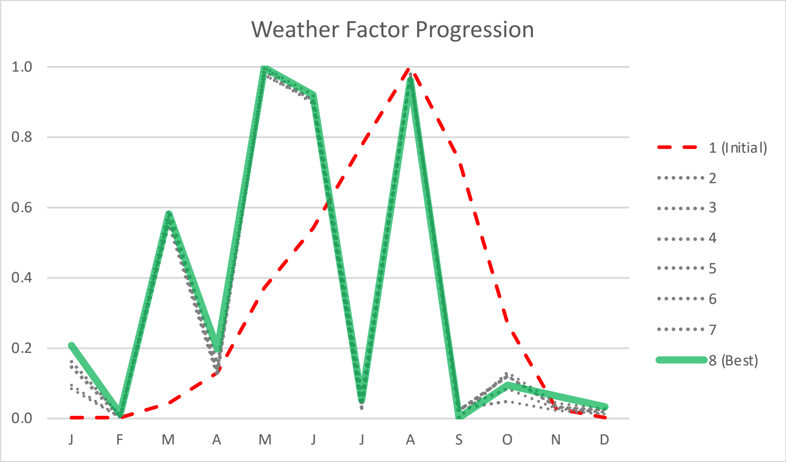

EF-S3: Infectivity decreases as annual rainfall accumulates. The table details the parameter optimization process for the EF-S1 model. *LHC* indicates the best sample from Latin Hypercube

sampling, *IPCP* indicates the results from the infectivity parameter calibration procedure (EF parameters fixed), and *APCP* indicates the results from alternating parameter calibration procedure. The rightmost columns indicate the number of replications required for RP and the resulting average WMSE. The remaining figures depict the fit for the IPCP and APCP parameters, as well as the change in EF distribution throughout APCP before convergence.

**Source**	*minRate*	*maxRate*	*inflection point*	*para*	*otherInt 2014*	*otherInt 2015*	*otherInt 2016*	**Reps for RP**	**Average WMSE from RP**
APCP	5.243e-06	1.162e-03	13.16	0.0735	0.3520	0.5312	0.3428	556	426.53
IPCP	6.934e-06	1.045e-03	10.05	0.0741	0.3145	0.4573	0.2990	766	538.52
LHC	1.000e-04	8.000e-04	25	0.0500	0.3500	0.4000	0.3000	2984	881.23

Results after IPCP (100 replications)

Number of worms exuded from dogs

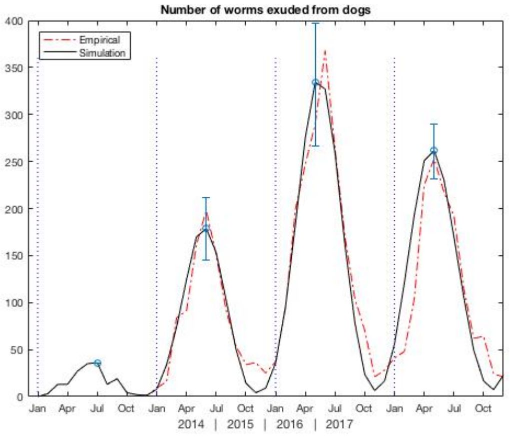

Number of dogs exuding worms

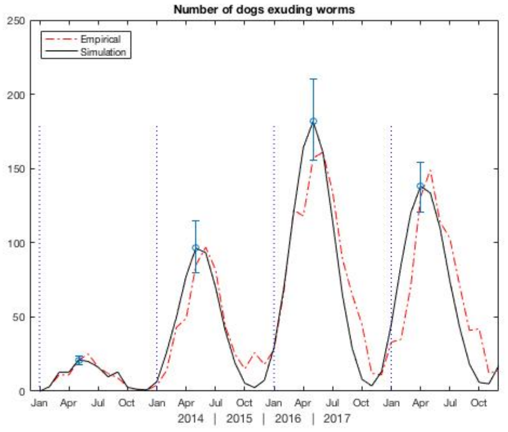

Results after APCP (100 replications)

Number of worms exuded from dogs

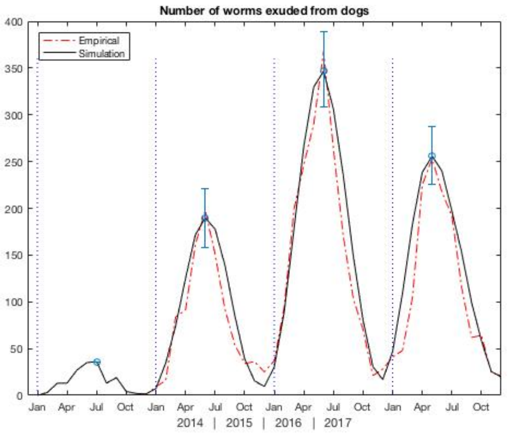

Number of dogs exuding worms

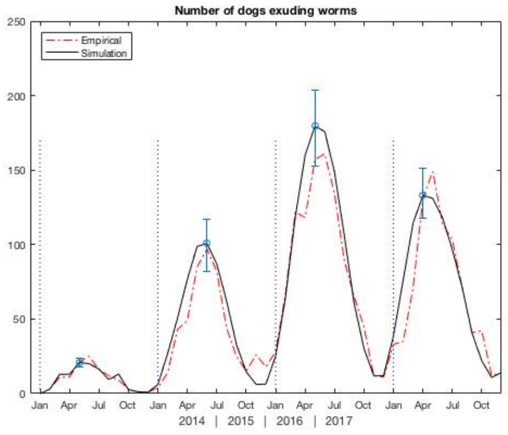

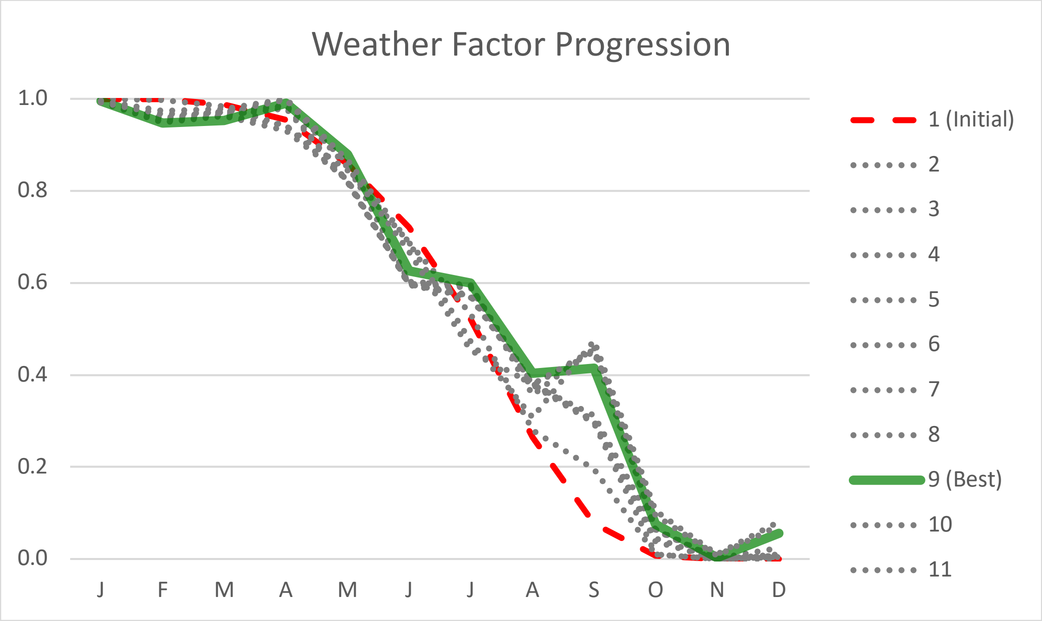

EF-S4: Infectivity is greatest before the rainy season and when temperature is high. The table details the parameter optimization process for the EF-S1 model. *LHC* indicates the best sample from Latin Hypercube sampling, *IPCP* indicates the results from the infectivity parameter calibration procedure (EF parameters fixed), and *APCP* indicates the results from alternating parameter calibration procedure. The rightmost columns indicate the number of replications required for RP and the resulting average WMSE. The remaining figures depict the fit for the IPCP and APCP parameters, as well as the change in EF distribution throughout APCP before convergence.

**Source**	*minRate*	*maxRate*	*inflection point*	*para*	*otherInt 2014*	*otherInt 2015*	*otherInt 2016*	**Reps for RP**	**Average WMSE from RP**
APCP	2.972e-04	1.992e-03	108.44	0.0791	0.5803	0.1940	0.1234	123	352.76
IPCP	2.904e-04	1.722e-03	94.09	0.0766	0.5163	0.2413	0.1994	1666	444.68
LHC	3.000e-04	1.750e-03	100	0.0800	0.4000	0.2000	0.2000	5000*	514.52

Results after IPCP (100 replications)

Number of worms exuded from dogs	Number of dogs exuding worms
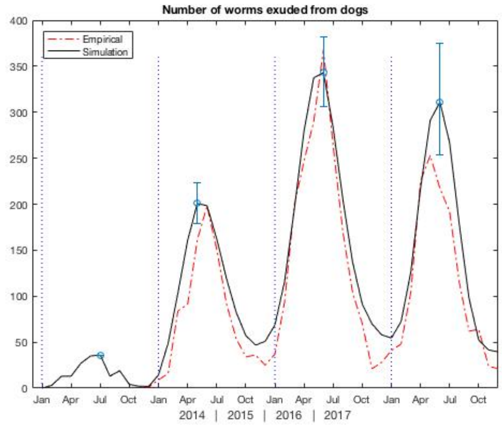	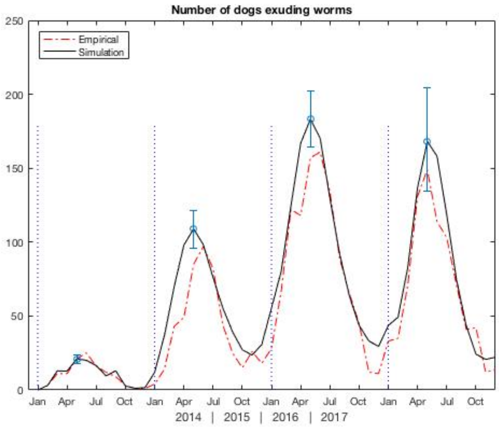

Results after APCP (100 replications)

Number of worms exuded from dogs	Number of dogs exuding worms
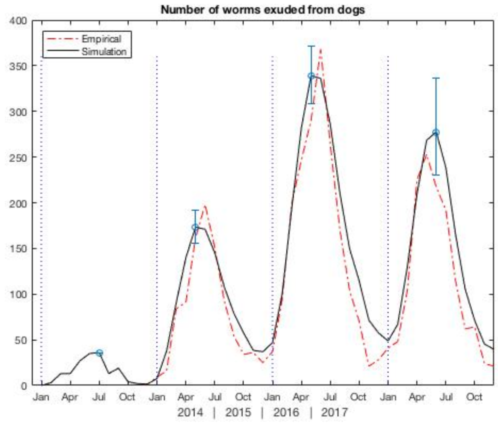	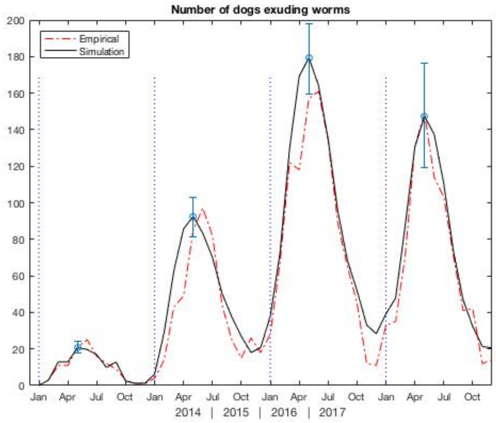

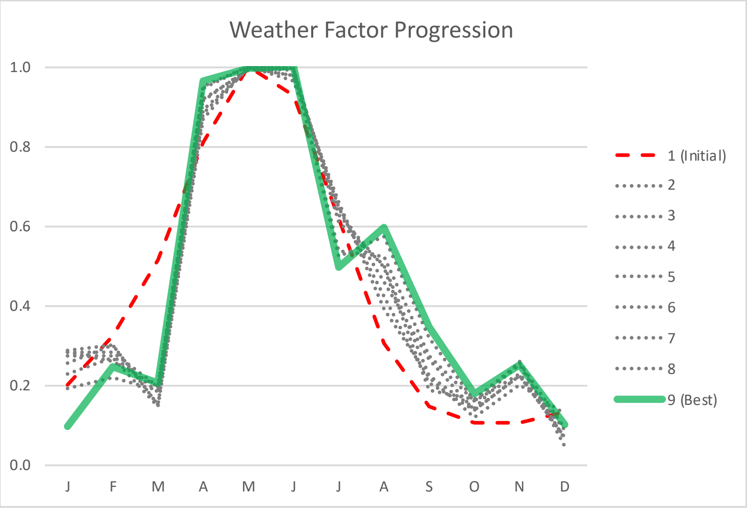

3.2) Identifying LHC samples that perform well across EFs.

We note that the calibrated parameters for EF-S1 and EF-S3 are quite similar because the local search during each of their calibration was initiated from the same initial sample of parameter values. When further comparing the best-performing initial samples from the set of 100 LHC samples (Appendix Section 3.1), we observed the following: with the exception of EF-S0 (which was always ill-fit), a small set of six samples performed well across all EFs. The fact that a select few samples perform well across multiple EFs suggests that these initial parameter values are successfully approximating a function for probability of infection that is rather *robust* to external, e.g. environmental, influences.

The following six samples from LHC sampling performed well for several different EFs (excluding EF-S0). In Table 7, we label the six samples A-F, indicate whether it is in the top 5 samples for the EF scenarios (denoted by "Yes" in the cell), indicate whether it is the top sample (denoted by the asterisk), and give the minimum and maximum of each of the parameter values.

Table 9. LHC samples that perform well across several EF distributions.

**Sample**	F_L	F_U	D	C	B_{2014}	B_{2015}	B_{2016}	*EF-S1 Top 5?*	*EF-S2 Top 5?*	*EF-S3 Top 5?*	*EF-S4 Top 5?*
A	2e-04	1.50e-03	400	0.01	0.30	0.00	0.00	Yes	Yes	Yes	Yes
B	2e-04	1.00e-03	10	0.02	0.50	0.00	0.35	Yes	Yes	Yes	Yes
C	3e-04	1.75e-03	100	0.08	0.40	0.20	0.20	Yes*	Yes		Yes*
D	4e-04	6.00e-04	40	0.01	0.50	0.20	0.00	Yes	Yes*		
E	5e-05	1.25e-03	50	0.07	0.00	0.00	0.20	Yes		Yes	Yes
F	1e-04	8.00e-04	25	0.05	0.35	0.40	0.30			Yes*	Yes
Minimum	5e-05	6.00e-04	10	0.01	0.00	0.00	0.00				
Maximum	4e-04	1.75e-03	400	0.08	0.50	0.40	0.35				

3.3) Worm burden in the water reservoir.

Every day, the number of (developed L3) worms in the water reservoir, n, is considered one day's *worm burden*. This daily worm burden is accumulated (in sum) over each month to determine the month's worm burden. At the end of the simulation, every month has an accumulated burden (not separated by year), which is divided by the overall worm burden to give a proportion of worm burden per month. All experiments are run for 100 replications. We do this for the 3 calibrated EF distributions that perform well after alternating calibration, i.e. EF-S1, EF-S3, and EF-S4. Results are shown in Table 8.

Table 10. Proportional worm burden per month, where months with 8-10% worm burden are indicted in orange and months with greater than 10% worm burden are in red.

EF	**J**	**F**	**M**	**A**	**M**	**J**	**J**	**A**	**S**	**O**	**N**	**D**
S1	2.1%	2.3%	5.1%	9.9%	13.7%	16.5%	15.9%	13.0%	8.7%	6.3%	4.0%	2.4%
S3	0.7%	2.0%	5.4%	9.8%	13.6%	16.4%	16.4%	14.0%	10.3%	6.6%	3.3%	1.4%
S4	2.1%	2.2%	5.1%	9.4%	13.4%	15.7%	15.4%	13.1%	9.0%	7.0%	4.6%	3.0%

3.4) Sensitivity analysis.

Given uncertainty in the literature about (i) the duration of L3s in the copepod host (interval [B] in Figure 2; see literature review in Appendix Section 1.4) and (ii) the maturation period of GW within the definitive host (interval [C] in Figure 2; see literature review in Appendix Section 1.5), we performed systematic analysis of our simulation model to test robustness with respect to the following two modeling assumptions (results are given in Appendix Section 3.4).

(A1) The life expectancy of each L3 in the copepod host is 30 days.

(A2) The randomized incubation period of each GW within a host is uniform 10-14 months.

(A3) All L3s are transmitted through a single water source.

For testing (A1), we replaced the fixed 30 day duration with various alternative distributions: in particular, a randomized duration of Uniform(0,30) days (with mean of 15 days), Uniform(0,60) days (with mean of 30 days) and a combined distribution, which is piecewise such that 80% of worms have duration of Uniform(0,30) days and 20% of worms survive up to twice as long with duration of Uniform(30,60) days (with mean of 18 days). The cases where larvae live up to 60 days could also be representative of the effect of a transport host, such as a fish or tadpole. The model output, compared to the empirical data, are displayed in Figure 6. The results, measured by WMSE of the fit, are summarized in Figure 7. Observe that simply randomizing the duration period while keeping the mean at 30 results in very similar output in average with just slightly greater variance. However, when larvae have average duration significantly shorter than 30 days, the simulation underestimates the fourth-year peak, and therefore the WMSE is significantly greater. If it is found that GW larvae duration in the water is shorter than 30 days, on average, the simulation should be recalibrated.

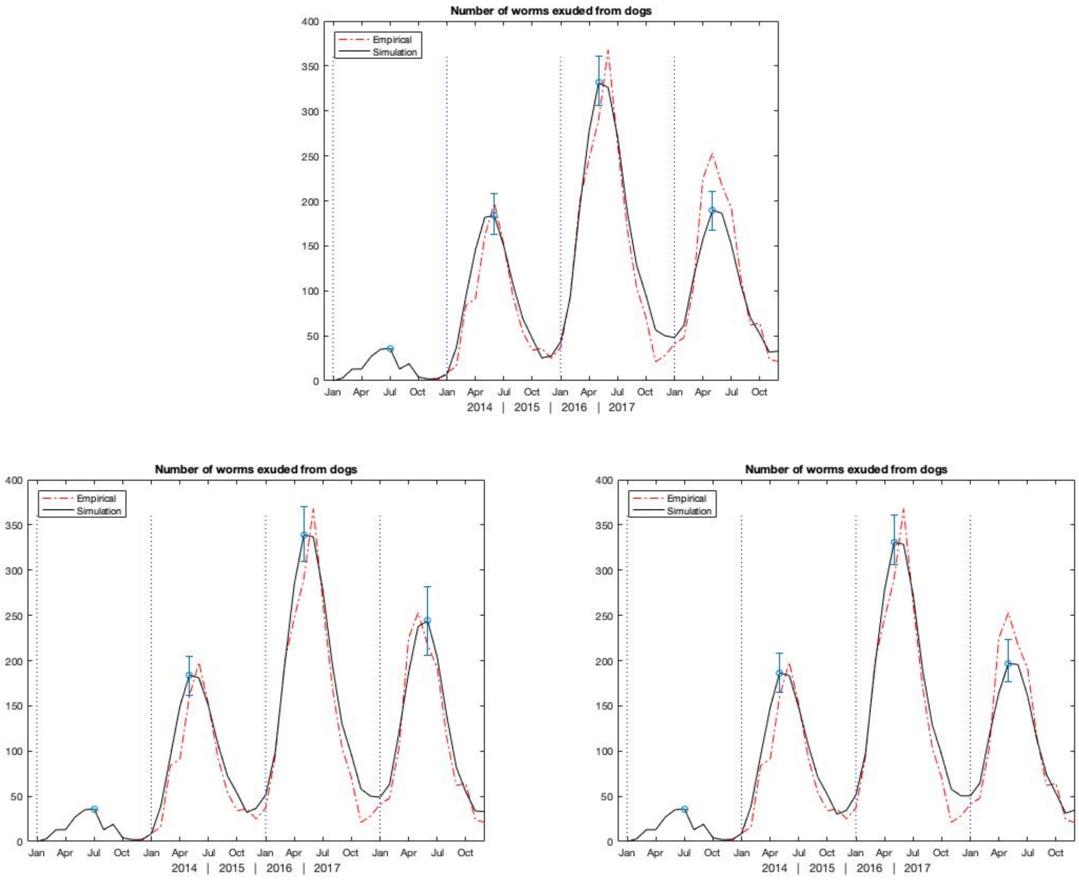

Figure 5. Model output (black) compared to empirical data (red) for two alternative duration periods. (A) Top: Uniform(0,30) days. (B) Lower left: Uniform(0,60) days. (C) Lower right: 80% with Uniform(0,60) days and 20% with Uniform(60,120) days.

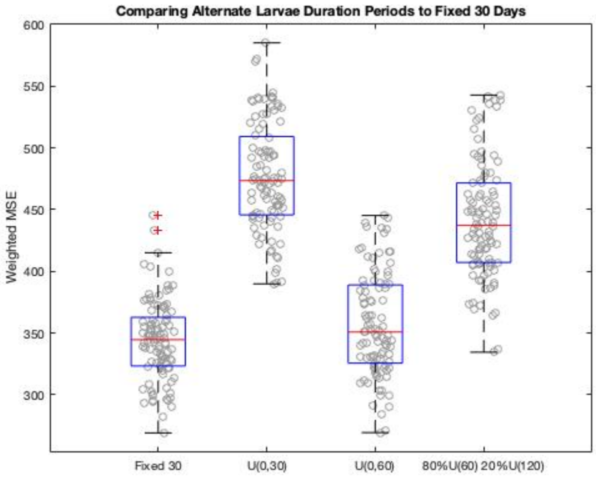

Figure 6. Comparison of WMSE over 100 replications of alternative duration periods.

For testing (A2), we compared the Uniform(10,14) distribution to the following alternatives: Triangular(10,12,14), Triangular(11,12,13), Uniform(10,12), and Uniform(12,14). We ran the best-known model, i.e., with calibrated EF-S2 infectivity and EF parameters, but simply replaced the incubation period for 100 replications. The model output, compared to the empirical data, are displayed in Figure 8. The results, measured by WMSE of the fit, are summarized in Figure 9. The model is relatively robust to changes in the *shape* of the distribution as long as the bounds and mean remain unchanged; however, the model is sensitive to changes in the bounds, e.g., Tri(11,12,13), and even more sensitive to shifts in the mean, e.g., U(10,12) and U(10,14). Furthermore, it is worse to *overestimate* the incubation period than it is to *underestimate* it, as shown by U(12,14) performing much worse than U(10,12).

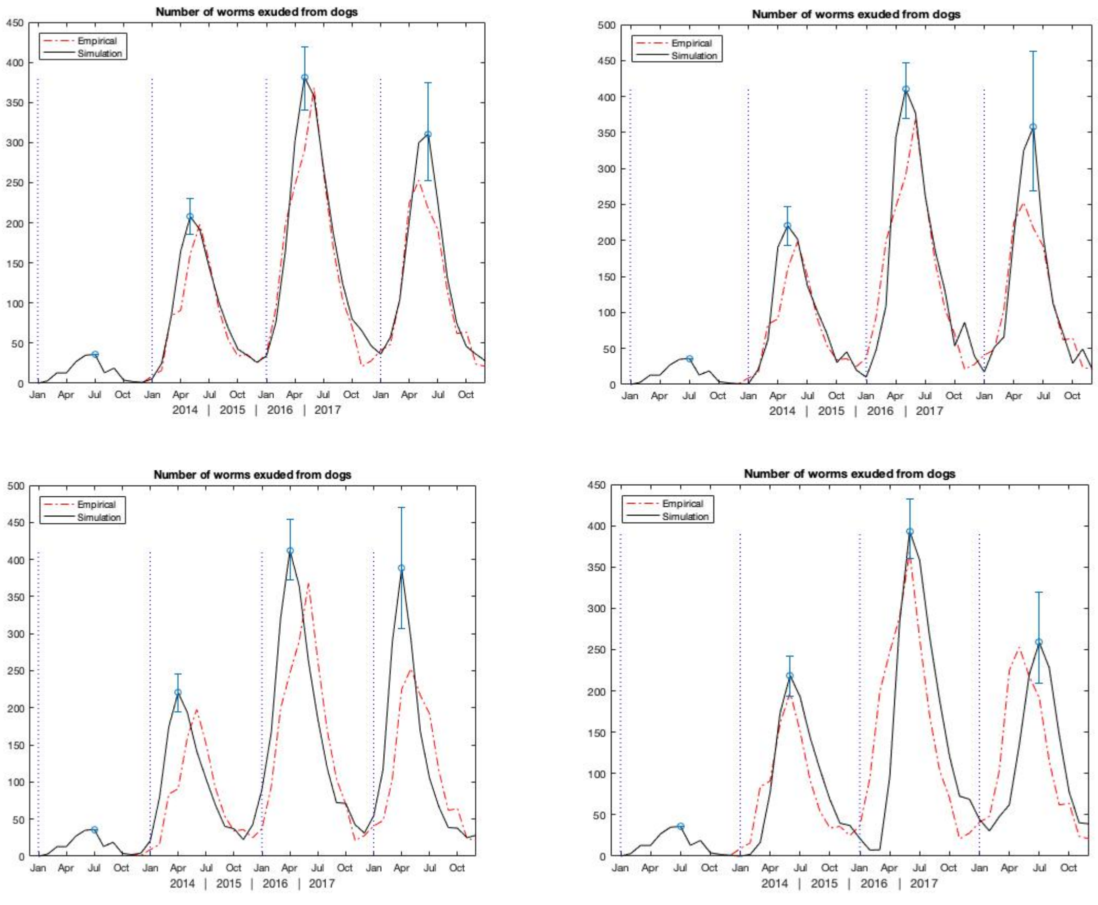

Figure 7. Model output compared to empirical data for four alternative incubation periods. (A) Top left: Triangular(10,12,14). (B) Top right: Triangular(11,12,13). (C) Bottom left: U(10,12). (D) Bottom right: U(12,14).

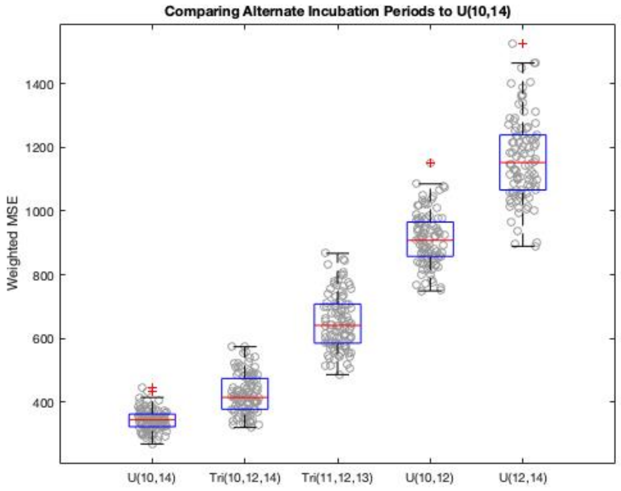

Figure 8. Distributions of WMSE for each alternative incubation periods.

For testing (A3), we introduce a second water source, each which may harbor L3s for transmission of GW, and allow for a proportion of dogs to have access to both. Note, these results are from the same parameters as published in the paper without recalibrating. First, we observed that with two water source model, the EF-S0 (no seasonality) assumption does not explain the consistent seasonality, just as in the single water source model. In general, the EF-S4 (temperature and rainfall) assumption performs best for the double water source model, as shown in Figure 9. Note that the peak level of infections is slightly lower in 2017 for the double water source model compared to the single water source model, which could likely be corrected by parameter calibration; however, the overall seasonality fits well, as is. In general, one would expect the number of water sources to affect the magnitude of transmission, not the seasonality.

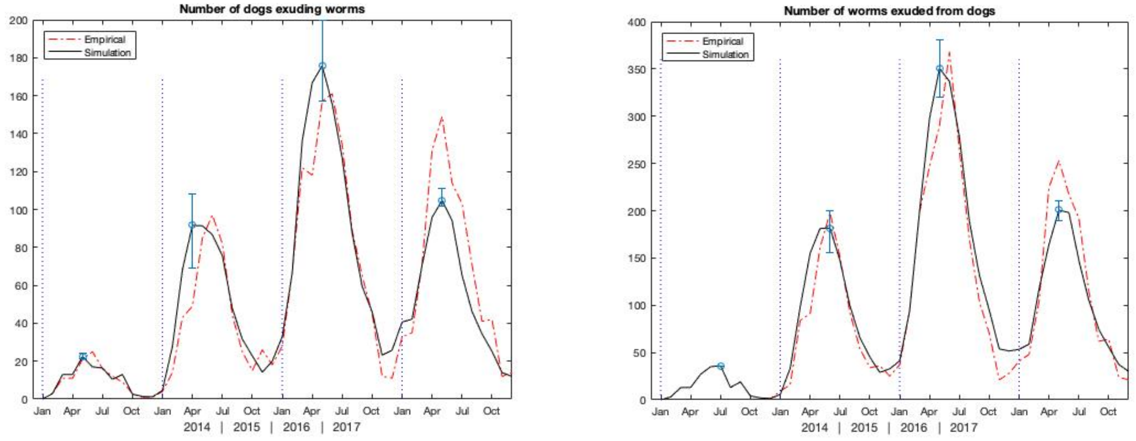

Figure 9. Two water source model with 40% Abate applied to both water sources.

3.5) Rationale for decreasing infectivity from April to September.

We have a number of theories for what could explain the declining infectivity in the transition from dry season to wet season, when water sources are limited and just beginning to be replenished. Intensive research is required to validate or refute these theories.

- Human activities, such as farming or fishing, are seasonally-driven and dogs frequently join their owners in many of these activities;
- Dogs (and humans) are restricted to fewer water sources, so they become concentrated locales for infection events (i.e., depositing larvae into a water source or consumption of larvae from an infected source);
- Since the volume of a water body is low, the density of "particulates" in the water is high and easier to consume in greater quantities, i.e., GW larvae consumed by copepods and copepods consumed by other hosts;
- As the volume of their water environment decreases, bottom-dwelling aquatic life, which may include copepods or some fish, become more easily accessible to land life;
- While dogs in Chad usually roam near their owner's home, the range of their movement is likely to decrease as the rain increases, which may be averting exposure to external (e.g., wildlife) reservoirs of infections; and
- Any correlated population dynamics of intermediate, paratenic, or transport hosts (e.g., a reduction in the number of tadpoles as they metamorphize) along the Chari river could point to an alternate route of transmission.

REFERENCES

1. Climate Data Online. 2018. https://www7.ncdc.noaa.gov/CDO/cdoselect.cmd?datasetabbv=GSOD. Accessed Jan 21, 2018.
2. Muller R. Dracunculus and dracunculiasis. *Adv Parasitol.* 1971;9:73-151.
3. Ruiz-Tiben E, Hopkins DR. Dracunculiasis (Guinea worm disease) eradication. *Adv Parasit.* 2006;61:275-+.
4. Cairncross S, Muller R, Zagaria N. Dracunculiasis (guinea worm disease) and the eradication initiative. *Clin Microbiol Rev.* Apr 2002;15(2):223-+.
5. Eberhard ML, Ruiz-Tiben E, Hopkins DR, et al. The peculiar epidemiology of dracunculiasis in Chad. *The American journal of tropical medicine and hygiene.* 2014;90(1):61-70.
6. Carlotti F, Nival S. Molting and Mortality-Rates of Copepods Related to Age within Stage - Experimental Results. *Mar Ecol Prog Ser.* Aug 1992;84(3):235-243.
7. Netshikweta R, Garira W. A Multiscale Model for the World's First Parasitic Disease Targeted for Eradication: Guinea Worm Disease. *Comput Math Methods Med.* 2017;2017:1473287.
8. Smith RJ, Cloutier P, Harrison J, Desforges A. A Mathematical Model for the eradication of Guinea worm disease. *Understanding the Dynamics of Emerging and Re-Emerging Infectious Diseases Using Mathematical Models.* 2012:133-156.
9. Link K, Victor D. *Guinea worm disease (Dracunculiasis): opening a mathematical can of worms*, Citeseer; 2012.
10. Ghosh I, Tiwari PK, Mandal S, Martcheva M, Chattopadhyay J. A mathematical study to control Guinea worm disease: a case study on Chad. *Journal of biological dynamics.* 2018;12(1):846-871.
11. *Guinea Worm Wrap-up #253*: WHO Collaborating Center for Research, Training and Eradication of Dracunculiasis, CDC; March 9, 2018 2018.
12. Escudero DJ, Lurie MN, Mayer KH, et al. The risk of HIV transmission at each step of the HIV care continuum among people who inject drugs: a modeling study. *BMC Public Health.* Jul 25 2017;17(1):614.
13. Alizon S, van Baalen M. Transmission-virulence trade-offs in vector-borne diseases. *Theor Popul Biol.* Aug 2008;74(1):6-15.
14. Regoes RR, Ebert D, Bonhoeffer S. Dose-dependent infection rates of parasites produce the Allee effect in epidemiology. *P Roy Soc B-Biol Sci.* Feb 7 2002;269(1488):271-279.
15. Paul REL, Bonnet S, Boudin C, Tchuinkam T, Robert V. Aggregation in malaria parasites places limits on mosquito infection rates. *Infect Genet Evol.* Sep 2007;7(5):577-586.
16. McKay MD, Beckman RJ, Conover WJ. Comparison of three methods for selecting values of input variables in the analysis of output from a computer code. *Technometrics.* 1979;21(2):239-245.
17. Rinott Y. On two-stage selection procedures and related probability-inequalities. *Communications in Statistics-Theory and methods.* 1978;7(8):799-811.
18. Gosavi A. Simulation-based optimization. *parametric optimization techniques and reinforcement learning.* 2003.

19. Goldsman D, Nelson BL. Comparing systems via simulation. *Handbook of simulation: Principles, methodology, advances, applications, and practice.* 1998:273-306.
20. Boesel J, Nelson BL, Kim S-H. Using ranking and selection to “clean up” after simulation optimization. *Operations Research.* 2003;51(5):814-825.
21. Chen C-H, Lin J, Yücesan E, Chick SE. Simulation budget allocation for further enhancing the efficiency of ordinal optimization. *Discrete Event Dynamic Systems.* 2000;10(3):251-270.
22. Nelder JA, Mead R. A simplex method for function minimization. *The computer journal.* 1965;7(4):308-313.
23. Pillai N, Craig M, Dokoumetzidis A, Schwartz SL, Bies R, Freedman I. Chaos synchronization and Nelder-Mead search for parameter estimation in nonlinear pharmacological systems: Estimating tumor antigenicity in a model of immunotherapy. *Prog Biophys Mol Biol.* Jun 19 2018.
24. Darmani Kuhi H, Shabanpour A, Mohit A, Falahi S, France J. A sinusoidal function and the Nelder-Mead simplex algorithm applied to growth data from broiler chickens. *Poult Sci.* Jan 1 2018;97(1):227-235.
